# Perspective of Fe_3_O_4_ Nanoparticles Role in Biomedical Applications

**DOI:** 10.1155/2016/7840161

**Published:** 2016-05-12

**Authors:** Mohammad Reza Ghazanfari, Mehrdad Kashefi, Seyyedeh Fatemeh Shams, Mahmoud Reza Jaafari

**Affiliations:** ^1^Department of Material Science and Engineering, Ferdowsi University of Mashhad, Mashhad 9177948974, Iran; ^2^Biotechnology Research Center and Nanotechnology Research Center, School of Pharmacy, Mashhad University of Medical Sciences, Mashhad 917751365, Iran

## Abstract

In recent years, although many review articles have been presented about bioapplications of magnetic nanoparticles by some research groups with different expertise such as chemistry, biology, medicine, pharmacology, and materials science and engineering, the majority of these reviews are insufficiently comprehensive in all related topics like magnetic aspects of process. In the current review, it is attempted to carry out the inclusive surveys on importance of magnetic nanoparticles and especially magnetite ones and their required conditions for appropriate performance in bioapplications. The main attentions of this paper are focused on magnetic features which are less considered. Accordingly, the review contains essential magnetic properties and their measurement methods, synthesis techniques, surface modification processes, and applications of magnetic nanoparticles.

## 1. Introduction

**Figure 1:**
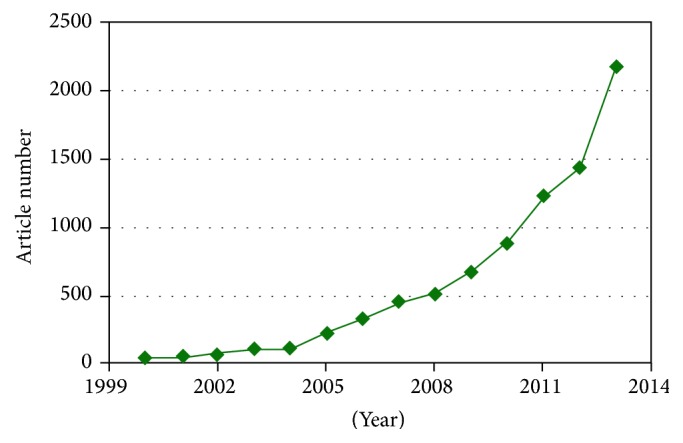
The increasing number of articles related to biomedical applications of magnetic nanoparticles based on Elsevier database.

For long years, the magnetic materials have attracted increased attentions as suitable candidates for use in differing applications due to their considerable performance [1]. On the other hand, by developing the nanotechnology in the last decades, the magnetic nanoparticles have found the special importance in the modern purposes like biomedical sciences caused by their unique characteristics [1–3]. Based on Elsevier database, Figure 1 shows the increased intendancies of scientists to work on these topics in recent decade.

Typically, iron oxides nanoparticles which are known as one of commonly used compounds for these applications can be formed in three natural types such as hematite (*α*-Fe_2_O_3_), maghemite (*γ*-Fe_2_O_3_), and magnetite (Fe_3_O_4_) [4]. Although all of these forms exhibit some promising properties like the biocompatibility and relatively low toxicity in human body, less sensitivity to oxidation, more stability in magnetic response (even upper to 50 years), possibility of transfer to superparamagnetic form by particle size decreasing, and ease of synthesis process and surface treatment, the magnetite nanoparticles have more appropriate behaviors [5–11]. For instance, in order to show suitable efficiency in several biomedical uses such as targeted drug delivery and contrast agent materials in magnetic resonance imaging (MRI), the nanoparticles should be exposed to maximum value of saturation magnetization (*M*_s_) and minimum value of coercive field (*H*_c_) and remnant magnetization (*M*_r_); thereby, owning to higher *M*_s_, the magnetite nanoparticles can be superior candidates [12–15]. Usually in most biological applications, the dispersity of magnetic nanoparticles in the fluid carrier has been imposed because of development of mobile particles system [16]. In order to achieve this aim, the dispersed ultra-fine magnetite particles should be stabilized in aqueous or other organic fluids [17, 18]. Generally, the fabrication of magnetic fluid is divided into two steps: the first step is the magnetic nanoparticles synthesis process and the second one is the stabilization of particles in the fluid by physical or/and chemical surface treatments [19–27]. The synthesis of nanoparticles can be done by coprecipitation [28], microemulsion [29], thermal decomposition [25], and hydrothermal [30], sonochemical [20], sol gel [31], and other different methods. Moreover, for the stabilization of particles in fluid and more biocompatibility of their surfaces, the employment of electrostatic dispersion agents [32] and various coatings (organic [33–35] or/and nonorganic/polymeric [36–39]) is widespread.

Historically, the primary studies on magnetic materials role in biological and medical applications were carried out after the discoveries about biomagnetism by Heinz Lowenstam in the 1960s [40]. Furthermore, for the first time, the magnetic fluids were prepared with magnetite nanoparticles at 1960 by NASA [41]. As a result of continued researches on these applications, nowadays the utilization of magnetite nanoparticles has become a useful approach in medical sciences such as targeted drug delivery [42, 43], contrast agent in MRI [44, 45], magnetic hyperthermia [46, 47], cell separation, and DNA detection [48]. Generally, in order to choose the best candidate for these applications, several important features should be considered, that is, the magnetic properties, particles size and morphology, and toxicity of selected materials [5–11].

The main aims of present paper are the investigation of essential magnetic properties and their measurement techniques, various synthesis methods, and different surface modifications of magnetite nanoparticles and the review of these particles' role in the medical applications.

## 2. Magnetite Nanoparticles

Magnetite is the one of iron oxides known as an oldest magnetic material and also called black iron oxide, magnetic iron ore, and loadstone [4]. Magnetite mineral crystallized with Fe_3_O_4_ chemical formula in spinel structures can exhibit strongest magnetism among other phases of iron oxides. Typically, the natural magnetite with density of 5.2 g/cm^3^ can be found as fine grains in sedimentary rocks with hardness of 5.5 to 6.5 in Mohs scale [4].

### 2.1. Crystal Structure

**Figure 2:**
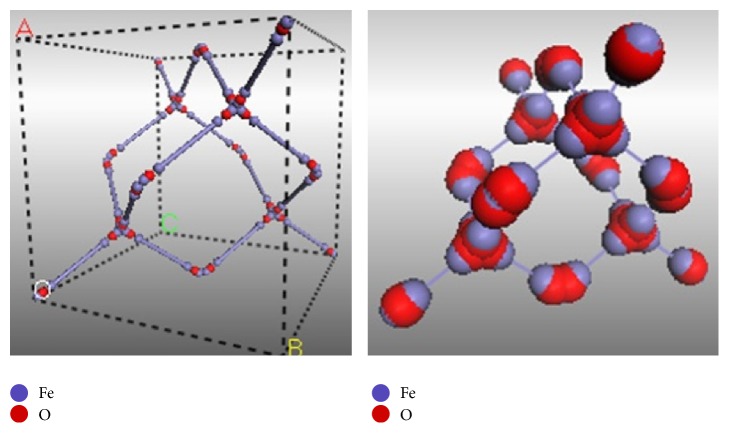
The polyhedral and ball models of magnetite crystal structure.

For the first time, the magnetite crystal structure had been studied in 1915 as one of the first mineral structures evaluated through X-ray diffraction method by Bragg and Nishikawa. In 1979, Hill et al. found that the magnetite structure is a reverse spinel (MgAl_2_O_4_) [49]. The unit cell of magnetite is face centered cubic and contains 32 close packed oxygen ions, crystallized in 8 formula units per unit cell of space group $Fd\overset{-}{3}m$ with structural parameter equal to 0.839 nm [4]. Simultaneously, the spinel structure of magnetite includes both Fe (II) and Fe (III) ions that are located at two sublattice sites with tetrahedral and octahedral coordination according to special order [50]. The Fe (III) ions completely occupy the eight tetrahedral sites and are randomly distributed in octahedral sites with Fe (II) ions [4, 51]. In fact, the tetrahedral sites only contain Fe (III) ions while the octahedral sites apply both of Fe (III) and Fe (II) ions [52]. Although in normal magnetite structure with stoichiometric formula of Fe^2+^_*x*_Fe^3+^_*y*_O_4_, the ratio of *x*/*y* is equal to 0.5 [4, 50, 51, 53, 54]; sometimes the Fe (II) ions can be completely/partially substituted with other divalent metal ions by change of the unit cell length parameter (*a*), which caused formation of metal ferrites [55]. In the magnetite unit cell, the atomic position parameters of Fe_1_, Fe_2_ (16d), and O (32e) atoms are “8a, 0.1250, 0.1250, 0.1250,” “16d, 0.5000, 0.5000, 0.5000,” and “32e, 0.2549, 0.2549, 0.2549,” respectively [56]. The polyhedral and ball models of magnetite crystal structure are shown in Figure 2.

Since the magnetic spins of octahedral and tetrahedral sites are opposite in direction and create two sublattice sites, thus, based on these two sublattice directions, the magnetite structure is ferrimagnetic [4, 49–51]. Various exchange interactions can occur between iron ions in both intra- and intertetrahedral and octahedral sites due to presence of these different crystal sites [53, 54]. In addition, in the magnetite structure, the Fe-O bonds length can be calculated based on the fractional atomic position of oxygen atoms (*u*(O)) and lattice parameter (*a*), according to valence sum rules [54]. Hence, these bonds lengths in tetrahedral and octahedral sites are equal to $\sqrt{3}\;\;a(u-1/8)$ and *a*(1/2 − *u*), respectively, while *u* = 0.255 for Fe_3_O_4_ [56].

### 2.2. Magnetic Properties

The various materials can show the wide range of magnetic behaviors such as paramagnetic, ferromagnetic, ferrimagnetic, antiferromagnetic, and superparamagnetic properties. Generally, in paramagnetic materials, the magnetic moments are randomly aligned while the external magnetic field is absent; thereby the overall net magnetization is zero. On the other hand, under the magnetic field, these moments are partially or even completely oriented to the field direction and indicated the sensitive net magnetization [4, 55, 57]. In the ferromagnetic crystals, all of the magnetic moments are coupled to each other, are aligned to the applied external field, and maintained this parallel order even after elimination of field [57].

**Figure 3:**
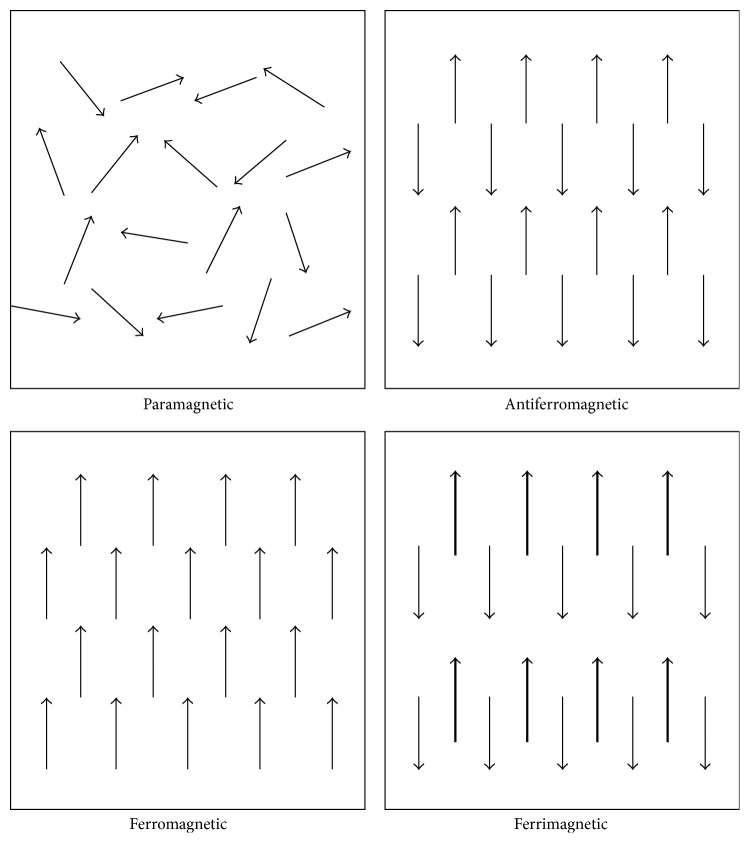
The statement of moment orientation in different magnetic structures such as paramagnetic, ferromagnetic, ferrimagnetic, and antiferromagnetic structures [60].

The magnetic materials with the similar antiparallel moments that show no net magnetization are called antiferromagnetic [4, 57]. Moreover, in the ferrimagnetic materials, although the alignments of moments are antiparallel, the net magnetization is not equal to zero because the vectors lengths of these moments are different [57–60].  Figure 3 shows the statement of moment orientation in each magnetic position [60].

As we know, based on the electronic configuration of iron atoms, there are four unpaired electrons in their “3d” orbitals. Accordingly, the magnetic behaviors of iron compounds are caused by activity of these itinerant electrons as exchange (between magnetic atoms like Fe-Fe), double exchange (between atoms of same element with different valences like Fe^2+^-Fe^3+^), and super exchange (between magnetic and nonmagnetic atoms like Fe-O) processes in the atomic energy bands range [58, 59]. The broadening of these ranges depends on the interatomic separation between the atoms, so that the width of bands is strongly reduced by increasing interatomic separation values [59].

**Figure 4:**
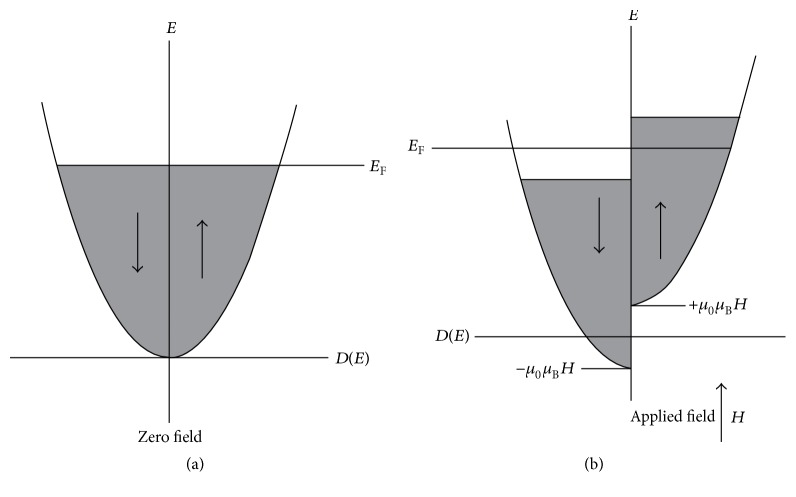
The schematic explanation of Fermi energy model of magnetic properties of materials. In (a) position, the up/down spins are located in balance states before applying external field. In (b) position, by applying external field, the energy state of aligned spins is higher than other ones; so, the magnetic properties are created [60].

One of the most useful models for the explanation of magnetic properties in these materials suggests that when the density of electron states, *N*(*E*), remains in the certain range, the number of situated electrons in the “3d” orbitals and also direction of their spins (up or down) can determine the kind and magnitude of magnetic behaviors [59]. Furthermore, the other influential parameters in this model are the effective exchange energy (*U*_eff_) of paired 3d-electrons (the energy of spins direction switching) and the density of state at the Fermi level (*E*_F_) [58, 59]. Thus, when both of these parameters are located in higher levels, the transfer of electrons to the magnetically stable state is increased. The schematic explanation of this model can be seen in Figure 4 [60].

#### 2.2.1. Ferrimagnetic Properties

In the bulk status, the magnetite structures show ferrimagnetic properties while the magnetic moments with congruent spins are oriented to the applied field direction. However, the alignment of moments is partial; hence, by these means, the actual magnetization has fewer amounts than theoretical one (summation of all moment magnetization) [59]. In fact, the bulk magnetite structures contain some magnetic domains where each one has independent moment vector with different magnitudes and directions. Therefore, owing to the vectors of domains with opposite arrangement that are canceled by each other, the total magnetization level of particles is attenuated [58, 59]. In the presence of multidomains structure, not only are the different orientations of moments caused, but also the alignment of moments to the applied field is more difficult due to blocking role of domain walls as barriers [60]. Typically, the magnetite particles in the size range of few microns to submicron scales (about 100 nanometers) indicate the ferrimagnetic behavior [60].

Moreover, the magnetite particles magnetization is gradually decreased by growth of temperature because of magnetic moments disordering based on thermal agitation rising [4, 60]. Consequently, whereas the temperature goes up to critical point (for ferrimagnetic materials called Curie temperature (*T*_C_)), the moment ordering is completely vanished; so, the breakdown of magnetization is occurred [58–60]. Among common magnetic materials, the ferrimagnetic particles of magnetite have relatively high Curie temperature equal to 850 K.

**Figure 5:**
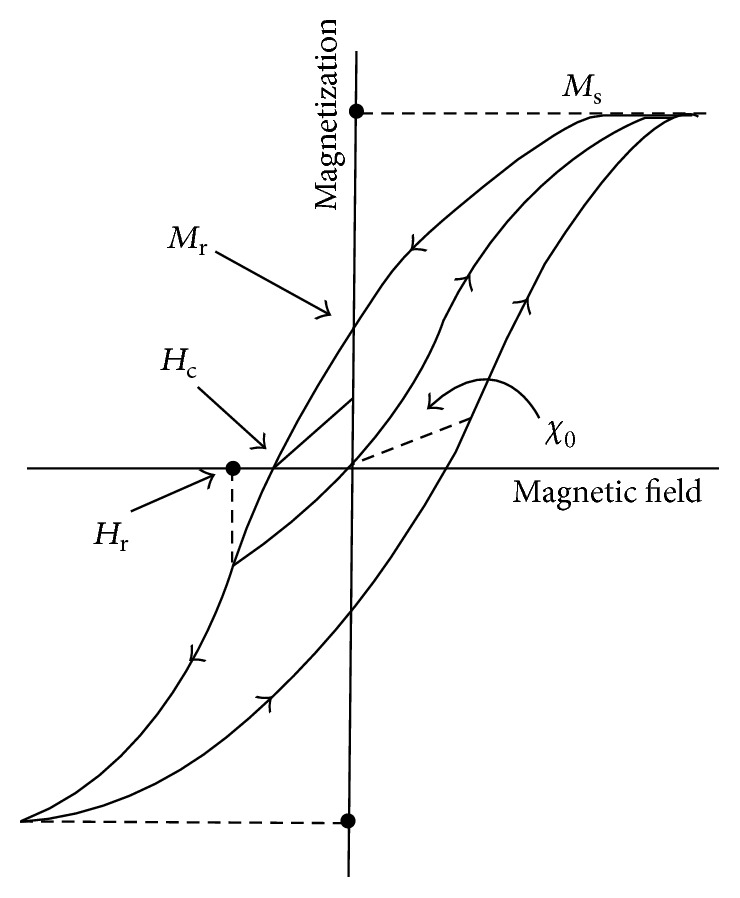
The magnetic hysteresis loop of ferromagnetic and ferrimagnetic materials. In this figure, several critical parameters such as saturation magnetization (*M*_s_), remnant magnetization (*M*_r_), and coercive field (*H*_c_) can be seen.

Figure 5 shows the magnetic hysteresis loop of ferromagnetic and ferrimagnetic materials that was given in detail by Weiss over 100 years ago [71]. In this figure, several critical parameters can be seen such as saturation magnetization (*M*_s_), remanent magnetization (*M*_r_), and coercive field (*H*_c_). Saturation magnetization is the maximum amount of magnetization due to dipole alignment to the applied field direction so that *M*_s_ go down by the decreasing of external field magnitude [55]. In ferromagnetic materials, even by removal of magnetic field, a little amount of magnetization remains which is called remnant magnetization and its value depends on the structural, microstructural, and compositional parameters of materials [55, 60]. Moreover, coercive field is the required field with opposite direction to eliminate remnant magnetization [55].

#### 2.2.2. Superparamagnetic Properties

In 1930, Frenkel and Dorfman offered a theory about the change of magnetic structures during the decrease of particle size [72]. They believed the ferromagnetic materials can transfer from multidomains to single-domain state by particle resizing to nanoscales. In the subsequent researches, this idea gradually developed and finally in the 1960s, the superparamagnetism theory was suggested [72–77]. Therefore, two phenomena occur in ferromagnetic nanoparticles; the first one is the creation of single-domain structure and the other one is the development of superparamagnetic behavior.

As mentioned before, ferromagnetic materials contain special magnetic domains where each one has a net magnetic moment. These domains are disparted by domain walls which have individual energy levels (*E*_W_). Considering that the magnetic moments within domains are desired to interact with each other, the amounts and situations of domain walls as barriers can affect the interactions (exchange); thereby the magnetostatic energy (*E*_M_) is created owning to these interactive behaviors [55, 76, 78]. Thermodynamically, domains system is preferred to keep the energy level in lowest mode by balancing between *E*_W_ and *E*_M_. On the other hand, *E*_W_ gradually grow up by increasing wall relative area due to decrease of particle size, so that if the particle size falls down below the critical point, the domain walls energy enlarges more than magnetostatic energy and their stability is lost; thereupon, the particles are formatted in single-domain magnetic structures [78, 79]. Usually, depending on materials characteristics, the range of critical size is experimentally obtained. Accordingly, the critical size of transformation to the single-domain mode at ambient temperature is expressed as follows:

(1) $$\begin{array}{c} D_{\text{c}}=P\frac{{\left(AK_{\text{u}}\right)}^{1/2}}{\mu_{0}{M_{\text{s}}}^{2}}, \end{array}$$

where there is assumption of spherical particles without interaction, *D*_c_ is the critical diameter of particles, *P* is the dimensionless constant that approximately equals 18, *A* is the exchange constant, *K*_u_ is the uniaxial anisotropy constant, *μ*_0_ is the magnetic permeability in vacuum state, and *M*_s_ is the saturation magnetization [78, 80]. According to the literature, not only are the critical ranges different in various materials, but also the different critical sizes have been reported for the magnetite particles. For instance, in several papers *D*_c_ amount of Fe_3_O_4_ particles at room temperature has been given greater than 100 nm, while most researchers believe this critical range must be less than 50 [81–90]. These distinctions could occur due to the experimental measurement errors and different effective parameters, whereas *μ*_0_ is the materials characteristic but *M*_s_, *A*, and especially *K*_u_ depended on external factors like synthesis method affecting structure and microstructure of materials.

**Figure 6:**
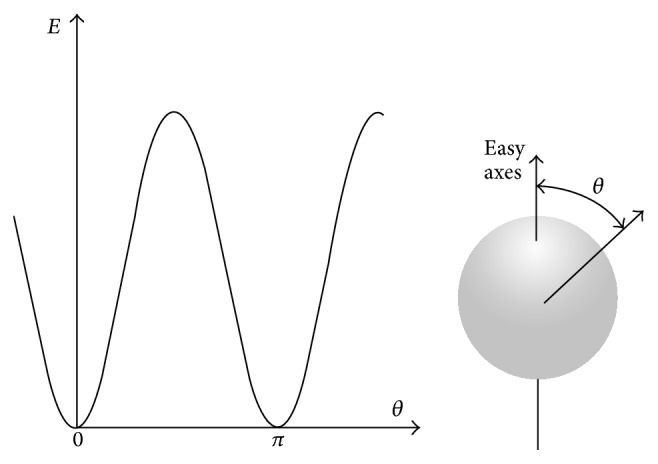
The schematic of variation of anisotropy energy as a function of moment vector directions. In easy axes (in this figure: 0 and *π* angles) the anisotropy energy is minimum and in hard axis (*π*/2) it is maximum amount [60].

The exchange direction of magnetic moments in multidomains materials can determine the state of internal energy; thus both of the easy and hard directions are defined in that the summation vector of moments is preferred to parallel to easy axes. In fact, the anisotropy energy is the energy difference of easy and hard axes that emerges by two phenomena: the spin-orbit interactions and the long range moments coupling [76]. Furthermore, by rising of crystal symmetry, the value of anisotropy energy decreases in the range of 10^2^ to 10^7^ Jm^−3^ in which this amount for magnetite particles is about 2 × 10^4^ Jm^−3^ [55]. The following equation is presented in order to calculate anisotropy energy:

(2) $$\begin{array}{c} E_{\text{A}}=KV\sin^{2\;}\theta , \end{array}$$

where *E*_A_, *K*, *V*, and *θ* are the anisotropy energy, effective uniaxial anisotropy constant, particle volume, and the angular difference between easy axis and summation vector of magnetic moment (magnetization), respectively. Assuming that the anisotropy energy acts as a barrier against the orientation of moment vectors, two easy axes can be defined in *θ* = 0 and *π* where the anisotropy energy is equal to zero at these angles. Moreover, the anisotropy barrier has a maximum value at *π*/2 angle as a hard axis. The schematic of variation of anisotropy energy as a function of moment vector directions is shown in Figure 6 [60].

**Figure 7:**
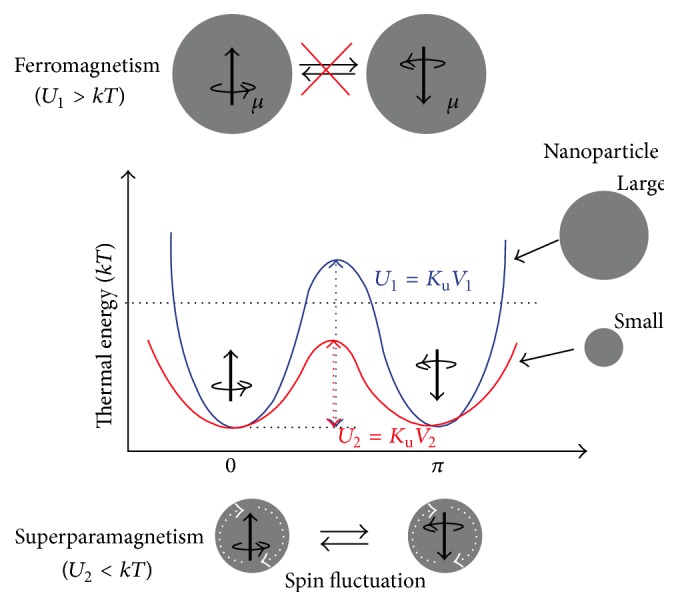
The variation of thermal and anisotropy energies by particles resizing from large scale (ferromagnetic structure) to fine nanoscale (superparamagnetic structure) [92].

On the other hand, the thermal energy leads to the fluctuation of magnetic moments as follows:

(3) $$\begin{array}{c} E_{\text{K}}=k_{\text{B}}T, \end{array}$$

where *E*_K_ is the thermal energy (kinetic energy), *k*_B_ is the Boltzmann constant, and *T* is the temperature (kelvin degree). Hence, the ferromagnetic particles can be adopted in two modes. First state occurred when the particle size is larger than a critical threshold; thus the particle volume (*V*) and anisotropy energy have relatively high amounts. In this state, the anisotropy energy overcomes the thermal energy and the magnetization vector prefers to align to the easy axes. The other mode happened while the particle size, particle volume, and anisotropy energy are gradually decreased, so that the anisotropy energy becomes less than thermal energy. Additionally, the magnetization vector can orientate to all directions. In fact, all directions have the same rules like easy axes; thereby the ferromagnetic particles prefer to behave like paramagnetic materials. Forasmuch as the structure is single domain (domain-wall-free), the system shows the superior paramagnetic behavior that is called superparamagnetism [78]. Moreover, for estimation of probability of magnetization alignment to the different directions, a formulation model has been suggested in several researches [91]:

(4) $$\begin{array}{c} f\left(\;\theta \;\right)d\theta =\frac{e^{\left(-E\left(\theta \right)/K_{\text{B}}T\right)}\sin\left(\theta \right)d\theta }{\int_{0}^{\pi /2}e^{\left(-E\left(\theta \right)/KT\right)}\sin\left(\theta \right)d\theta }, \end{array}$$

where *f*(*θ*) is the probability of magnetization alignment to each angle, *K*_B_ is the Boltzmann constant, and *T* is the temperature. Accordingly, if the thermal energy (*K*_B_ *T*) is greater than anisotropy energy, the probability of magnetization standing along easy axes (*f*(*θ* = 0)) is small. On the contrary, the magnetization can be deviated from the easy axes while the thermal energy is comparable to the anisotropy energy. The schematic of these changes is seen in Figure 7 [92].

**Figure 8:**
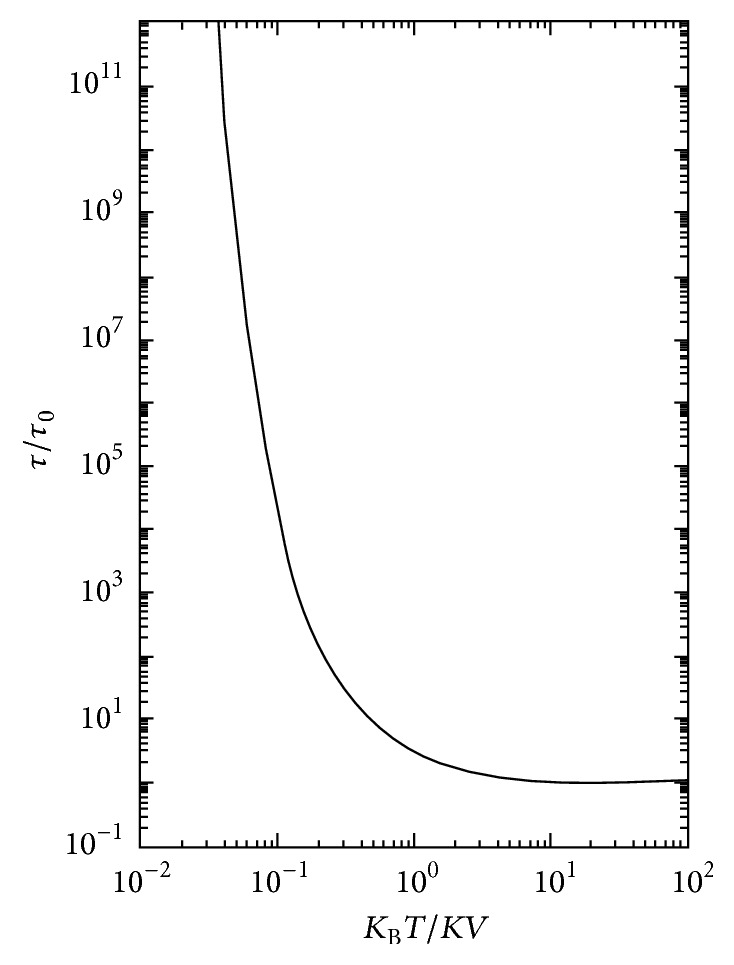
The variation of relaxation time as a function of thermal energy to anisotropy energy ratio values.

One of the most important characteristic behaviors of superparamagnetic materials is the required time for deviation of magnetization from easy axes that is called relaxation time. This parameter is stated by Neel-Brown equation as follows [55, 58, 76, 78, 91, 93, 94]:

(5) $$\begin{array}{c} \tau =\tau_{0}e^{\left(K_{\text{u}}V/K_{\text{B}}T\right)}, \end{array}$$

where *τ* is the relaxation time, *τ*_0_ is the characteristic time (inverse of attempt frequency) that is equal to 10^−9^ to 10^−12^ seconds, *V* is the volume of particle, *K*_u_ is the anisotropy energy, *K*_B_ is the Boltzmann constant, and *T* is the temperature. The amount of *τ*_0_ depends on some parameters such as temperature (weakly dependence) [82, 95], particle size, saturation magnetization, gyromagnetic ratio, and different kinds of anisotropy [96–100].  Figure 8 shows the variation of relaxation time as a function of energy ratio values [76].

As can be seen in Figure 8, in higher energy ratio the relaxation time is very short and vice versa. On the other hand, the experimental techniques for measurement of relaxation time have considerable limitations; therefore, the minimum quantifiable time period is called experimental measuring time (*τ*_*m*_). Consequently, the critical temperature has been defined as a criterion; hence, in higher temperatures than this amount, the quantity of energy ratio is large and the relaxation time (*τ*_0_) is lesser than *τ*_*m*_. In these situations, it is supposed that the system is superparamagnetic. In contrast, if the applied temperature is lower than critical amount, the relaxation time is greater than *τ*_*m*_ and the system behavior shows the deviation from superparamagnetism; so, this status is known as a blocking state [76, 78]. Moreover, the critical temperature that is called blocking temperature (*T*_b_) is presented by some various expressions in different literature depending on the measuring techniques [76, 78]. For the system of the similar size particles without interaction, the blocking temperature is obtained in this manner [76, 78]:

(6) $$\begin{array}{c} T_{\text{b}}=\frac{K_{\text{u}}T}{K_{\text{B}}\ln\left(\tau_{m}/\tau_{0}\right)}. \end{array}$$

**Figure 9:**
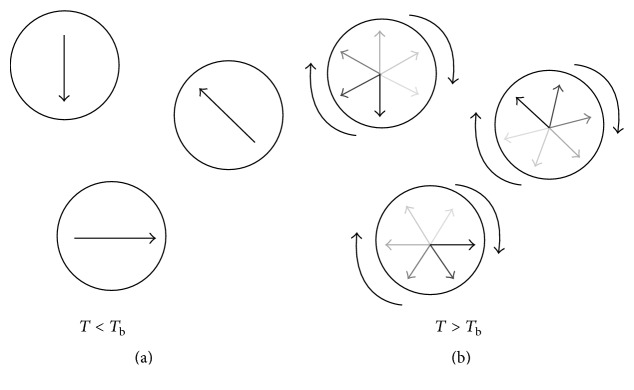
The details of superparamagnetic behavior as a function of blocking temperature and relaxation time. In state (a), temperature is below the blocking temperature and the anisotropy energy is dominated to the thermal energy; so, the moments are apparently fixed. In state (b), temperature is higher than blocking point and the thermal energy is larger than anisotropy one; thereby, the superparamagnetic structure is formed [95].

Figure 9 indicates the details of superparamagnetic behavior as a function of blocking temperature and relaxation time [95].

Blocking temperature depends on several parameters such as particles size, anisotropy constant, experimental measurement method, environment temperature, and applied magnetic field [78, 90]. The blocking temperature can be changed by varying of external field, so that, according to (7), *T*_b_ is reduced as a result of rising of field [90]:

(7) TbH=Tb01−HHck,

(8) Hc=2KuMs,

where *T*_b_(*H*) is the blocking temperature after applying field, *T*_b_(0) is the blocking temperature before applying field, and the *k* is the experimental constant that for low and high fields has been estimated to be equal to 2 and 0.66, respectively [101, 102].

In addition, the anisotropy energy included four parts such as magnetocrystalline, strain, shape, and surface anisotropy among which the crystalline and shape anisotropies have stronger roles in the nanoscale particles [76]. The importance of these kinds of anisotropy in design of different samples is more specifically described in Section 2.3.

#### 2.2.3. Measurement of Magnetic Properties

In order to efficiently study magnetic properties of materials such as magnetite nanoparticles, the utilization of useful measurement methods is necessary. Although many of these methods can be used to obtain wide range of magnetic parameters, the cognizing of limitations and sensitivities of each quantity is most helpful [103]. Generally, the most important magnetic properties can be divided into some classes such as magnetic moment (*m*), magnetization (*M*), magnetic field (*H*), magnetic flux density (*B*), magnetic polarization (*J*), magnetic Curie temperature (*T*_C_), blocking temperature (*T*_b_), and magnetic anisotropy (*K*). Some of these parameters are listed with their units in Table 1.

Typically, in order to determine the basic magnetic features of nanoparticles, the hysteresis loops have been drawn such as *M*-*H* curve (magnetization curve) for evaluation of intrinsic properties (e.g., effects of chemical composition and crystal structure) and *B*-*H* curve to simultaneously study the intrinsic and extrinsic properties (e.g., effect of samples shape) [60, 103]. According to the hysteresis curve results, some important information has been extracted like magnetic ordering of materials such as para-ferro-ferri-anti-ferro- and superparamagnetism, magnetic nature of materials (soft or hard), and magnetic power (*M*_s_) [60, 96]. As follows in this section, the common techniques to acquire magnetic hysteresis and other parameters have been presented. Furthermore, some of the significant errors of these methods have been expressed.

(*1) Vibrating Sample Magnetometer (VSM)*. One of the common choices for determination of hysteresis curve and related parameters in many researches is the vibrating sample magnetometer (VSM). This device includes some important parts such as “hall effect” sensors that can measure the intensity of magnetic field (*H*), strong external magnetic field that is created by superconducting magnets or water-cooled electromagnets, pick-up coils, and sample vibrating system [103]. In order to obtain magnetic moments by VSM, it is necessary that the external field has slow directional change; thus, in most instruments, the sample holders are usually vibrating while the pick-up coils are fixed [103]. With the purpose of magnetic moment measurement, the signals of sample vibration that is proportional to the magnetic moment, vibration frequency, and vibration amplitude are detected by coils and consequently filtration of frequency and amplitude effects is done by some complicated systems [104]. Furthermore, VSM can be used to study magnetic properties at very high and/or very low temperatures by employment of atmosphere controlled oven (to avoid sample oxidation) and liquid nitrogen or helium cryostats, respectively. Accordingly, by these ways, the evaluation of Curie temperature and blocking temperature (in superparamagnetic samples) is straightforwardly possible [103, 104]. Moreover, particles size can be determined by VSM according to the blocking temperatures results.

Generally, VSM has been used to study the hard and semihard magnetic materials by common pick-up coils and also soft magnetic materials by replaced Helmholtz coil systems [103–105]. Testing samples can include liquid or solid phases with bulk (through various shapes such as sphere and toroid), melt spun, powder, nanoparticles, and thin film forms with different natures such as metals, ceramics, or even biological specimens [103]. Typically, VSM is sensitive device to fast measurement of magnetic properties with resolution of 10^−9^ Am^2^ by used commercial pick-up coils and 10^−12^ Am^2^ by utilized SQUID sensors [106–108]. Furthermore, VSM can be used to study the magnetic anisotropy of materials while torque coils have been coupled with pick-up coils [103]. Moreover, as mentioned before, by VSM employment the measurement of blocking temperature is possible; thereby, the size of particles can be achieved by utilization of *T*_b_ amount according to the relationship equation of anisotropy energy and thermal energy (thermal agitation phenomena) [106]. This method can be used by other magnetometers like AFGM and SQUID.

For extended information about VSM method, refer to [103–110].

(*2) Alternating Field Gradient Magnetometer (AFGM)*. Similar to VSM, the alternating field gradient magnetometer can determine magnetic dipole moments of materials. Principles of this method include the measurement of mechanical force changing on piezoelectric sensors that is caused by alternating magnetic field gradient. The changing field gradient leads to sample vibrations that can be detected by piezoelectric sensors. Afterward, by piezosensors, the mechanical forces are converted to electrical signals that are proportional to the gradient amplitude and magnetic moments of specimen. In AFGM, considering high amount of signal to external field noise ratio (about 500), the measurement sensitivity is very high. Measurement resolution of AFGM is about 10^−13^ Am^2^ that means 1000 times more than commercial VSMs precision [103, 111, 112]. Additionally, like VSM, the measurement time is very quick and the study of properties at different temperatures is possible by utilization of oven and cryostat systems [111, 112]. On the other hand, main drawback of AFGM is the limitation of measurement sensitivity that is originated by acoustic and mechanical noises in environment [103].

Although AFGM can be used to determine the magnetic properties of samples with different shapes and size, the major ability of this method is assessment of thin films features [103].

(*3) Superconducting Quantum Interference Device Magnetometer (SQUID)*. Like VSM, the principles of SQUID magnetometer are based on sample vibration and detection of magnetic flux alternating, but with differences in coils type [103]. In SQUID, specimen is vibrated with frequency of 0.1 to 5 Hz that initiates a change in magnetic flux. After that, this change is determined by very sensitive fixed SQUID coils instead of common pick-up coils. Measurement resolution of SQUID is comparable to AFGM and about 1000 times more than VSM (about 10^−13^ Am^2^); thereby, it is suitable method to obtain the properties of samples with weak magnetic features [106]. Additionally, by employing oven and liquid N_2_ or He cryostat, SQUID can be used at various temperatures [103]. Main limits of SQUID include sensitivity of measurement to noises of external magnetic field, sensitivity of results to geometry of purposes, and very long measurement time (30 to 1200 min) due to point to point measurement system [113, 114]. SQUID has been utilized to survey different samples but especially to be handled to study nanomaterials and thin film [103].

(*4) Measurement Methods of Magnetic Anisotropy*. In many bioapplications of magnetic nanomaterials like magnetic hyperthermia, specimens must be magnetic anisotropy; it means that the required magnetic energy to dipoles rotation depends on directions. Therefore, it is necessary that anisotropy properties of samples are assessed obviously. In order to obtain these parameters, in addition to the above devices, some methods are identified such as torque magnetometer, singular point detection technique, and thermomagnetic measurement systems [103].

Torque magnetometers work based on recognition of sample torque that is caused by rotation of purpose to easy axes direction under intense magnetic field. Measured torque is commensurate to magnetizing energy, anisotropy energy, and anisotropy constant of samples [109]. The key challenges of this instrument are the need for single crystal as a purpose and sensitivity to mechanical noises. The most important applications of torque magnetometers include mensuration of nanoparticles and thin film anisotropy properties [103, 109].

The principles of singular point detection (SPD) technique are applied to determine the magnetizing energy in perpendicular and parallel directions to the external field. The anisotropy field (*H*_a_) can be clarified according to the difference of two measured amounts [115–117]. By SPD method, the study of magnetic anisotropy of polycrystals and composite powders is easily possible. In contrast, SPD can detect only the anisotropy field, and also, for proper functioning, the strong external field is required [118, 119].

Thermomagnetic systems are the analytical transformation method based on detection of time dependent magnetizing parameters in zero field cooling (ZFC) where has been claimed that it can be used to determine anisotropy and particle size of nanoparticles [103, 120, 121].

(*5) Other Methods for Specific Parameters*. Sometimes, in order to obtain several properties of magnetic materials, the utilization of different methods such as Helmholtz measuring coils, coercimeter, magnetoimpedance, and pulsed field magnetometer methods is so useful, although these systems are not common to study powder samples [103, 105].

On the other hand, Mossbauer spectroscopy is one of the most helpful techniques for investigation of chemical composition, formed bonding in structures, and magnetic properties of nanoparticles which works based on hyperfine technique that means creation of nuclear transition in samples by employment of gamma ray source [122–124]. In this method, due to different movement of ray source into the sample, the transition amounts can be varied between ground and excited energy levels that depend on the kind of interactions among sample nuclei and existing fields (electrical or/and magnetic) [123]. Accordingly, the study of natural and induced magnetic properties of particles can be carried out based on number of excited levels and intensity of excitation that have been explained by first and second Zeeman effects [123].

**Figure 10:**
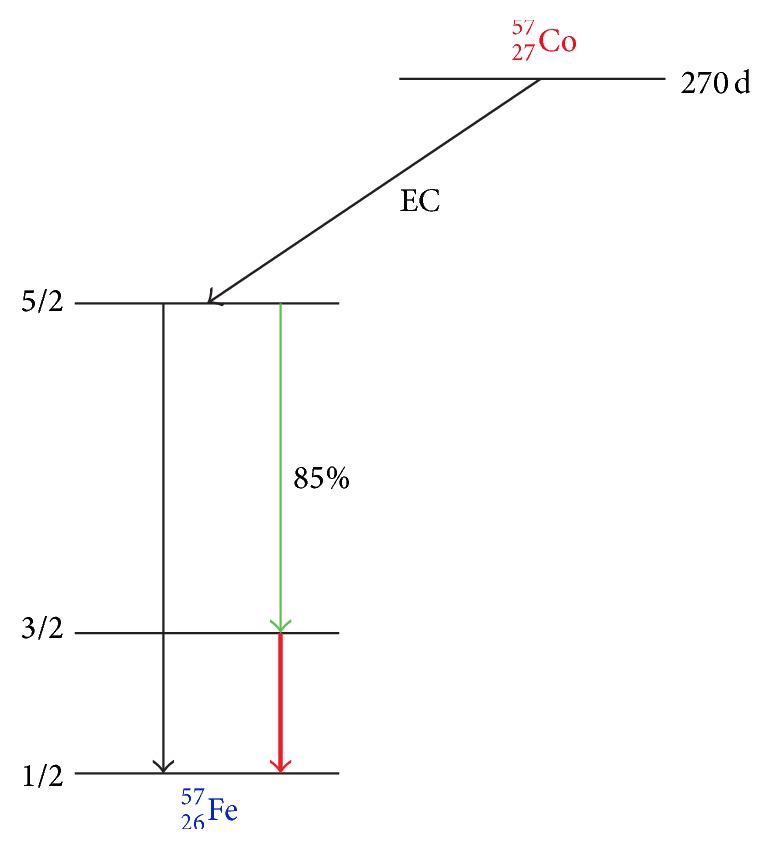
The principle of Mossbauer spectroscopy. The nuclear reactions in Mossbauer source cause the desired spectroscopy [123].

In Mossbauer spectroscopy, the selection of gamma ray sources depends on the samples compositions that have the same nuclear energies as each other. Furthermore, the selective source should have high mass and high Debye temperatures that lead to radiating low energy gamma ray [122–124]. For instance, the appropriate source to analysis of Fe^57^ compounds can be ingredient cobalt^57^.  Figure 10 shows the principle of Mossbauer effect on Fe samples, schematically [123]. On the other hand, the major drawback of Mossbauer spectroscopy is the limitation of applicable number of elements in periodic table [125].

Furthermore, when the measurement of independent elemental moments of samples is mainly important, the X-ray magnetic circular dichroism (XMCD) and polarized neutron reflectometry (PNR) methods can be used to measure the magnetic moments in each element separately, although these devices require complicated and nonsimply available facilities like synchrotron and neutron based systems [103, 125].

**Figure 11:**
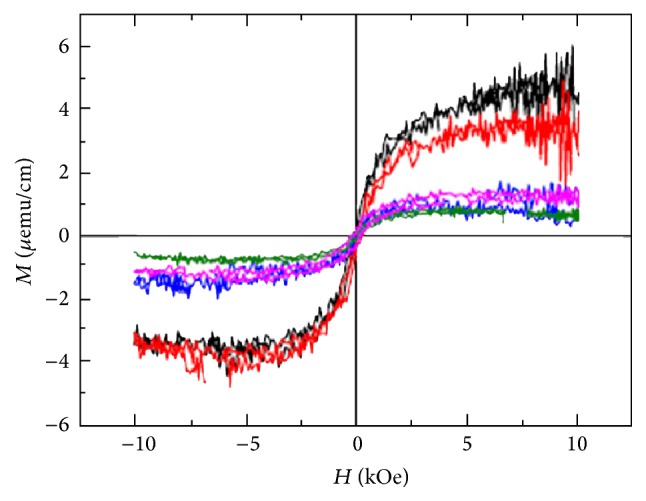
The disorder noises in *M*-*H* curves of nanoscale sample that are caused by utilization of diamagnetic substrate during measuring [126].

In following, the rough comparison of important parameters of measurement methods like measurable properties and sample conditions has been presented in Table 2.

(*6) Measurement Errors*. Although the measurement methods of magnetic properties are mostly developed, the study of magnetic nanoparticles has some complications due to measurement sensitivity of very small magnetic moments per atoms (nanomagnetic moment). Considering resolution of common magnetometer such as VSM, AFGM, and especially SQUID that can detect moment less than 10^−10^ emu, the characterization of nanomagnetic moment of ferromagnetic, ferrimagnetic, and superparamagnetic nanoparticles can be so susceptible [103, 105, 125]. According to literature, major errors of these techniques have been divided into double parts: the first one is the existence of magnetic contamination and the other one is the artifacts of methods (software or/and hardware) [103]. Magnetic contamination can be originated from some source such as sample handling, magnetic dust materials in laboratory atmosphere, sample preparation or modification process (especially by metallic tools), and effect of sample substrates and holders [103]. Generally, most of the contamination has low *H*_c_ and *M*_r_; so, their effects on the low-dimensional structures like superparamagnetic nanoparticles are considerable [125, 126]. Usually, sample substrates have diamagnetic properties that lead to observation of disorder noises in *M*-*H* curves as indicated in Figure 11 [126]. In order to remove this contamination, the utilization of acid cleaning (with proper damage of some samples) and employment of ultrasonic bath with acetone, deionized water, and propanol solution during some cycles can be more useful [126].

As mentioned before, another main source of errors comes from measurement devices that are including insufficient sensitivity of device to shape and size of particles (it is automatically fixed as a default) and disability to detect magnetic moment of each phase or element, independently (in common methods); so, magnetic properties of substrates can be detected as sample particles [103, 126].

**Figure 12:**
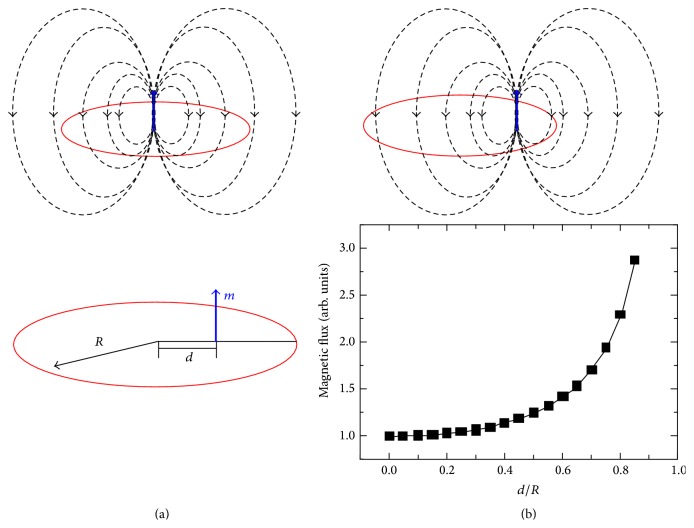
The effect of sample and coils positions on magnetic flux distribution. In case (a), the positions of sample and coil are determined by arrow and circle, respectively. Moreover, the sample distance from center point is defined by “*d*” symbol. In (b) case, it is clear that the imbalance position of sample and coil led to creating considerable errors in flux amounts [126].

Moreover, in study of magnetization and particularly magnetic anisotropy, some errors can occur by nonprecise position of sample holder and coils that are usually adjusted via manual controls [126, 127].  Figure 12 shows the effect of sample and coils positions on magnetic flux distribution that influences the amounts of *M*_s_ and anisotropy constants [126].

In order to distinguish main magnetic properties and errors, use of high or low temperature magnetometry like FC and ZFC is an appropriate method [126]. The principle of these methods is based on different temperature dependency level of magnetic properties of main sample and most of the contamination; for instance, blocking temperature (*T*_b_) and Curie temperature (*T*_C_) of diamagnetic substrates and ferromagnetic or superparamagnetic particles have considerable differences, frequently [126].

### 2.3. Particle Size and Morphology Effects

Usually, the magnetic properties of nanomaterials can be varied by increase of surface to volume ratio of particles and variation of their size.

Decreasing of particles size in nanometer scales led to creation of magnetic death layer on the particles surface due to deviation of magnetic moments orientation, formation of disordering configuration of moments that are caused to develop superparamagnetic behavior (below specific critical size), making a difference in normal cationic distribution in crystalline structure, and finally variation of some surface properties like ability of water absorption on surface [54]. Creation of superparamagnetic behavior not only can be guided to the decreasing of agglomeration probability of particles due to diminishing of coercive field (*H*_c_) and remnant magnetization (*M*_r_) amounts like paramagnetic materials, but also can show relatively high saturation magnetization (*M*_s_) quantity as well as ferromagnetic and ferrimagnetic materials (in bulk state) [128]. Different materials show various critical sizes to transform to superparamagnetic state. In fact, this critical size is characteristic of each composition.  Table 3 illustrates the critical size of some common superparamagnetic materials. Furthermore, decreasing of particle size and increasing of their surfaces are very useful for biological and medical applications. In contrast, when particles size is decreased less than 10 nanometers (for many of materials), the crystallinity of particles is also decreased, so the amount of saturation magnetization (*M*_s_) is dropped off [128]. Furthermore, the variation of particle size can affect magnetic anisotropy properties. As mentioned in Section 2.2.2, the anisotropy energy amount of particles depends on their volume, so by decreasing of particle size the amount of anisotropy energy is decreased. In addition, the shape of particles can play important role in magnetic anisotropy conditions called shape anisotropy [129, 130]. For instance, the shape anisotropy is equal to zero when the shape of particles is spherical completely. Moreover, the 30% oriental difference in diameters of single-domain iron particles can lead to increase in anisotropy energy up to 400%. In general, the reason of shape anisotropy is the magnetostatic field phenomenon that also can affect the creation of magnetic domains structure [129, 130]. Although in many of nanostructured materials the crystalline anisotropy is very important, the shape anisotropy has significant effect on total anisotropy energy especially in some applications such as heat generation based on loss mechanism in nanoferrofluid for hyperthermia or other applications [129, 130].

Furthermore, in bioapplications, and especially in vivo applications, the size of particles has high importance, because the smaller particles with homogenous morphology are more biocompatible and confirmed with immunologic systems of body [131, 132]. Also, according to low *H*_c_ and *M*_r_ amounts of these particles, they have less agglomeration probability after applying magnetic field; thereby, their movements in blood and excretion from body are easier than larger particles [131, 132].

## 3. Synthesis Methods

In many bioapplications, in order to achieve some properties like more homogenous distribution of constituent parts, the particles usually have been used in fluidic state that is called ferrofluid. These stable colloidal suspensions have features of both magnetic particles and fluidic carriers simultaneously [19–25]. Typically, in order to fabricate ferrofluid system, two methods have been known. In the first method, the ferromagnetic or ferrimagnetic materials heating reaches up to the melting phase. This method is feasible merely for cobalt-palladium alloy [133]. The other method that is very useful for most nanomaterials like magnetite includes two parts: the first one is the synthesis of magnetic nanoparticles with appropriate characteristics, and the second one is the stabilizing and distribution of nanoparticles in proper liquid carrier [134].

Historically, for the first time in the 1960s, the ferrofluid systems had been provided based on use of Fe_3_O_4_ particles by NASA researchers [41, 135]. According to literature, magnetite nanoparticles can be synthesized by different physical and chemical methods such as coprecipitation [4], microemulsion [49], sol gel [136], thermal decomposition [53], hydrothermal and sonochemical methods [51], and mechanical alloying technique [50]. Commonly, the chemical techniques have more advantages than physical ones, such as the possibility of synthesizing new materials with suitable properties, and more chemical homogeneity owning to combination of precursors in the molecular scales; thus the nanosize materials are directly produced [19–21]. Additionally, the chemical methods usually have capability of nanoparticles production in industrial scales [22, 23]. However, these methods have some drawbacks such as the toxicity of several precursors, formation of some surplus intermediate phases like iron hydroxyl, and probability of particles agglomeration during or/and after synthesis process. Common mechanism in most of chemical methods is the solid precipitation of target particles from supersaturation suspension of initial materials that has two stages [19–25]. Initially, the particles nucleate from solution as homogeneous or heterogeneous mechanisms, subsequently based on diffusion mechanism that can be controlled by concentration gradient and reaction temperature; the initial seeds are propagated up to desired size [19–21]. One of the important points in synthesis methods is the simultaneous nucleation of all seeds; thereby, the propagation step is carried out without any new nucleation that leads to the formation of smaller nanoparticles with narrow size distribution and lower probability of agglomeration [21–23]. In order to control and optimize the final properties of particles such as particle size, size distribution, crystal structures, and crystallinity degree, the significant knowledge about crystal chemistry, kinetics and thermodynamics of reaction, and phase equilibrium of materials is essential [19–25].

In the following, the widespread synthesis methods of magnetite nanoparticles are studied.

### 3.1. Coprecipitation

Due to simplicity and mass production ability in industrial scale, coprecipitation is the most common method for synthesis of magnetite nanoparticles [25]. In this technique, the initial solution including Fe^2+^ and Fe^3+^ salts is reduced with alkaline solution like NaOH and NH_3_·H_2_O. Usually, the reaction is designed with Fe^2+^/Fe^3+^ ratio equal to 1/2 that is sometimes partially varied in order to compensate for oxidation probability [137–139]. In general, coprecipitation method is applied in two parts such as (1) precipitation of iron hydroxides and (2) formation of iron ferrite based on the following equations [137]:

(9) M2++2Fe3++8OH−⟶MOH2·2FeOH3

(10) MOH2·2FeOH3⟶MFe2O4·nH2O+4−nH2O

According to literature, although the synthesized particles by coprecipitation have higher crystallinity degree than other methods, the size of these particles is relatively larger with wide size distribution which is not appropriate to use in many bioapplications [137, 138]. In order to improve these conditions, employment of experiment design methods and some theoretical models like Avrami model can be useful to control kinetic parameters such as temperature, pH, mixing rate, and integration rate of initial materials [139]. For example, the increasing of mixing rate can lead to faster nucleation and thereby formation of smaller particles [137–139].

As a magnetic view, because of more crystallinity and subsequently higher saturation magnetization, coprecipitation is the proper method for bioapplications in the event where size distribution is completely controlled in narrow range. In these situations, the growing of temperature in the range of 20 to 100°C can assist in increasing nucleation rate (due to supplement of activation energy) and crystallinity degree [139]. However, the excessive temperature rising caused coarsening of particles [137, 138]. According to some literature, one of the most common methods to control size distribution is the size sorting of particles in the additional process like Massart sorting methods [140–144]. In addition, in the recent years, many efforts have been focused on in situ size controlling mechanisms during coprecipitation process [145–147]. For examples, Wu and coworkers [148] synthesized the sub-5 nanometers magnetite nanocrystals by control of temperatures and times of coprecipitation reaction. Furthermore, the coprecipitation method that coupled with ultrasonic waves had been used by Cheraghipour and colleagues in order to fabricate Fe_3_O_4_ nanoparticles with narrow size distribution [149–152]. Moreover, in many researches, utilizing some functionalizing coats in order to prevent particles growth by coating of initial nucleus can be seen [145–147]. The effects of some different types of these coats that are called chelating agents like citric acid, lauric acid, and vegetable oils have been studied until now [153–156]. Moreover, the kind of precipitation agents and solutions can affect particle size, crystallinity, and magnetic materials. For instance, by employing NH_3_·H_2_O as precipitation agent and alcohol as reaction solution, the saturation magnetization has higher amount than the case of NaOH utilization in water solution [157].

### 3.2. Microemulsion

**Figure 13:**
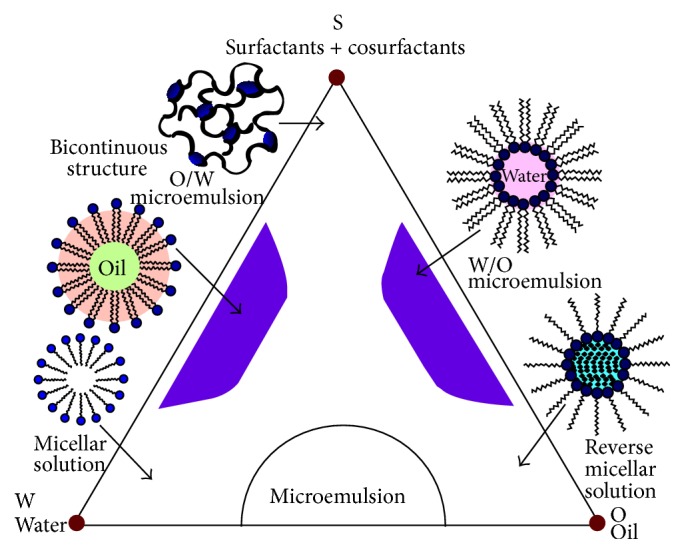
Theoretical ternary phase diagram of microemulsion systems [158].

Microemulsion is the novel method to synthesize different kinds of nanoparticles such as organic, metallic, and nonorganic nonmetallic materials that has attracted much interest in recent years [158]. Microemulsions, especially the reverse micelle methods (water in oil), are the appropriate techniques of magnetic nanoparticles formation due to large interfacial area, low interfacial tension, thermodynamic stability of immiscible solutions, and unique properties [158–160]. In these systems, owing to microemulsion dynamic and Brownian motion of micelles, they can collide with each other and lead to intermicellar exchange that is the main mechanism of the syntheses reactions [158–161]. Moreover, considering that the reactions are carried out in the nanoreactores called micelle, the size distribution of particles can be controlled in suitable narrow range [160–163]. According to literature, in order to synthesize magnetite nanoparticles by microemulsion, some parameters are impressive such as type of precursors, reaction temperature and time, and especially amounts ratio of water/oil/surfactant phases [158–163]. In fact, the relationships between these amounts have been represented as the ternary phase diagrams that can be very useful to control the final properties like size, distribution, morphology, crystallinity, and magnetic properties of particles when coupled with temperature effect [158–163].  Figure 13 shows schematically ternary phase diagram of water/oil/surfactant [158]. In order to find more details, the works of Malik et al. are so helpful [158].

Furthermore, the type of surfactants affected final properties and performances. Accordingly, Lu et al. studied the effect of different surfactants utilization such as anionic surfactants like SDS, cationic surfactants like DTAB and CTAB, and nonionic surfactants like Brij30 on the crystal defects, stoichiometric situations, and magnetic properties of Fe_3_O_4_ nanoparticles; hence, it has been concluded that although the size of magnetite particles is less than 16 nm in all cases, the cationic surfactant can lead to better magnetic properties like *M*_s_ and *H*_c_ for biological applications [160].

### 3.3. Hydrothermal

Among various processes to synthesize magnetite nanoparticles, the hydrothermal technique has significant ability of particles fabrication with very narrow size distribution [19, 20, 164]. The main reason of these properties is raised from use of high temperature in hydrothermal reaction that can affect nucleation kinetics [165]. Furthermore, for Fe_3_O_4_ synthesis by this method, sulfates and chlorides are used as cations source and water or ethanol is usually used as solution medium; thus, hydrothermal method is known as an environment friendly process [78, 166]. Due to employment of high temperature and particularly pressure in hydrothermal method, the relatively complex equipment like autoclave is required. Accordingly, some researchers have attempted to modify process, recently. For instance, Ahmadi et al. revealed modified systems to magnetite nanoparticles synthesis at low temperatures (140°C) without having to autoclave and have studied kinetics of reaction, although their magnetic properties are comparatively inadequate in short reaction time (below 2 h) [165]. In addition, the major drawback of hydrothermal technique is the long time consuming reaction at any given temperatures and thus the amounts of fabricated yields are low [167, 168]. In order to overcome this problem, one of the best approaches is the microwave assistant hydrothermal (*M*-*H*) method that can improve the kinetics of reaction up to two orders of amount by localized heating of solution [168]. Komarneni et al. have synthesized the magnetite nanoparticles with suitable magnetic properties by this process [169]. More details about *M*-*H* method have been found in Sreeja and Joy researches [168].

### 3.4. Thermal Decomposition

Thermal decomposition is one of the most effective synthesis methods of magnetite particles [164]. The main advantages of this method include excellent particle size controllability with narrow size distribution and high crystallinity of productions [53, 164]. Accordingly, the particles synthesized by thermal decomposition are very appropriate candidates for bioapplications like targeted drug delivery, magnetic hyperthermia, and MRI contrast agents [170–175].

The base of thermal decomposition methods is funded on the nucleation and growth steps of magnetite particles from initial materials in the high temperature processes. The precursors of this process are categorized into two classes, for example, the organometallic components of iron (such as Fe(acetylacetonate)_3_, Fe(N-nitrosophenylhydroxylamine)_3_, or/and Fe(CO)_5_) and the organic surfactant and solvents (such as fatty acid, steric acid, oleic acid, and hexadecylamine) [7, 35, 176–182].

According to the literature, the properties of synthesized powders depend on the process time, temperature, ratio of initial materials, kind of surfactants and solvents, and so forth [7, 35, 176–182].

Thermal decomposition process includes two steps: the first one is the nucleation of initial seeds by reaction of precursors and the next level is the propagation of nucleated seeds under reflux reaction at different temperatures and times [7, 35, 176–182]. Typically, the thermal decomposition method occurs from two approaches including the addition of hot solvents to reaction medium that can lead to fast nucleation process and the heating up of the reaction mixture up to decomposition temperatures. In the last approach, due to the relatively slow nucleation, the homogeneity of particles size and morphology is less than the first one [183]. The main disadvantages of this method include high required temperature (up to 300°C) for reflux process, complexity of long time consuming reaction, and depending on nonpolar organic solvents [184]. In recent years, many efforts have been focused on the modification of this process to achieve higher stability and magnetic properties of synthesized particles. Pérez-Mirabet and colleagues performed pot synthetic methods by use of oleylamine as a multipurposes agent, for the sake of stabilization of particles in solution (stabilization agent) and control of particles size (capping agent). By this method, they could achieve monodisperse particles with average size of 12 nm that showed appropriate amount of *M*_s_ equal to 76 emu/g [183]. Moreover, some researchers work on the solvent-free or/and biosolvents thermal decomposition methods that have more biocompatibility and less toxicity of precursors [185, 186].

In order to more accurately study synthesis methods based on the magnetic view, the key properties of synthesized particles by various methods are compared to each other in Table 4 [61–63, 78, 164].

## 4. Surface Treatments

Generally, in order to attain the ferrofluid systems with suitable properties for bioapplications, two critical parameters are most important. The first one is the stability of magnetite particles in solution. Due to the hydrophobic nature of particles surface and high surface area to volume ratio of particles, the synthesized nanoparticles tend to agglomeration to decrease this ratio. On the other hand, in bioapplications, it is necessary that the nanoparticles have been used with no agglomerations; for instance, in some bioapplications (in vivo ones) like targeted drug delivery and hyperthermia, the agglomeration of magnetic particles can lead to clogging the arteries and thereby dysfunction of application mechanisms [187]. Furthermore, the biocompatibility of particles is the milestone point in these applications. Accordingly, in order to achieve these aims, the utilization of appropriate coatings on particles is commonly used [188, 189]. These coatings can affect considerably the stability, biocompatibility, and especially magnetic properties of particles; hence, the proper coatings must cause no attenuation of magnetic properties [187]. Typically, the application of coatings can be carried out in two modes: during particles synthesis as an in situ process and after synthesis as an independent process. Although the in situ coatings are more complex, they can lead to providing the further stabilization and magnetic properties [187–189].

Based on literature, the used coatings are classified into two general types: organic and inorganic coatings that have been precisely investigated in the following sections.

### 4.1. Organic Coatings

Recently, the various types of organic materials have been widely utilized as proper coatings of magnetite particles owing to their unique properties, that is, biocompatibility, prevention of particles agglomeration by their surface modification to hydrophilic state, betterment of their dispersity by creation of electrostatic and steric stabilization methods, and particles functionalization ability by use of functional groups like hydroxyl, carboxyl, amino, and aldehydes [184, 187].

Typically, in order to coat the particles by organic materials, the variation of three important parameters of coated particles should be considered including magnetic properties, biocompatibility, and linking ability to other structures such as drugs, antibodies, proteins, enzymes, and DNA [184]. In many applications, the magnetic properties of modified particles are the major determinant factor of their performance; thus the utilized coatings must show minimum reduction in these properties. Although one of the most considerable disadvantages of some organic materials especially large molecular structures is the decrease of magnetic particles after coating process, these materials caused the creation of biocompatibility and particles linking to other additional structures [184, 187].

**Figure 14:**
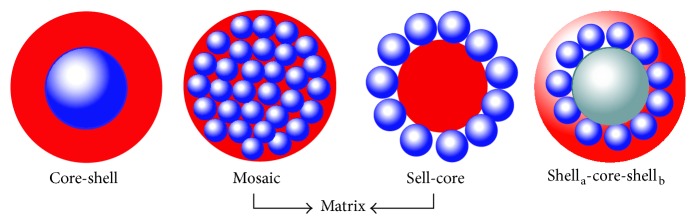
The schematics of organic coating models such as core-shell, mosaic, and shell-core structures [184].

Generally, the organic materials can coat magnetite particles as different models include core-shell, matrix, and core-shell-core structures [184]. In the core-shell structure, the magnetite particles act as core and organic materials have shell roles. Furthermore, this structure has a lower size and suitable dispersity relative to the other structures, and it is ideal model for magnetic particles with relatively high magnetic properties like magnetization [187–189]. On the other hand, while the magnetization of particles is low (for instance, due to low crystallinity), the matrix structure is more appropriate because in this model a greater number of particles are located in halo aggregation of coating materials, so the magnetization of these particles is associated together with higher amount [187–189]. Nevertheless, the matrix structure combined particles are formed in higher size (above 70 nm) compared to other models, commonly. Shell-core-shell structures are the specific models that are created based on usage of two different kinds of organic materials as shells. In these structures, usually, the size of production is similar to matrix model, but their magnetic properties are lower.  Figure 14 illustrates the schematics of these coating models [184].

According to the literature, the organic coating materials are categorized to three general classes, that is, small molecules and surfactants, macromolecules and polymers, and biological molecules.

#### 4.1.1. Surfactants

Surfactants (surface active agents) could improve the stability and dispersity (by decreasing the surface tension of the liquid), biocompatibility, and in some cases functionalization of particles in solutions [184]. Based on the nature of surfactants, they are divided into the three subclasses including oil soluble surfactants and water soluble and amphiphilic ones.

(*1) Oil Soluble Surfactants*. Oil soluble surfactants are the hydrophobic (lipophilic) groups that act as stabilization agents in oil-based solutions. In fact, the particles surface is hydrophobized by linking to these groups; thereby, the particles are considerably stabilized and their agglomeration is decreased [194–200]. The main components of oil soluble surfactants include the fatty acids like oleic acid and stearic acid, linear and/or branched alkyl phenol, hexadecane, phosphates, alkyl phosphonates, lauric acid, and dodecyl phosphonates [200–203]. Typically, by employing surfactants, the average size of particles is increased up to 5 nm and their saturation magnetization (*M*_s_) is changed negligibly [203–206]. Although these materials caused the good biocompatibility and suitable dispersity of particles, they have some disadvantages like their usability only in organic solutions (organophilic environments). Modified magnetic particles by oil soluble surfactants can be used in various bioapplications such as in vitro usages, MRI contrast agents, and drug carries in targeted drug delivery systems [194–206].

(*2) Water Soluble Surfactants*. Commonly, water soluble surfactants are utilized to transformation of hydrophobic nanoparticles to hydrophilic mode in aqueous solutions. The mechanism of this method is based on stabilization of particles by adding bipolar surfactants, that is, amino acid, citric acid, vitamin, and polyethylene glycol [207–211]. The functionalization by water soluble surfactants can be performed on the basis of two approaches. In the first one, the biocompatible bipolar agents have been added to the reaction medium during synthesis process. This one-pot method is most appropriate for creation of matrix structures. In fact, by adding excess amounts of surfactants, the coating aggregate particles are formed as matrix structures [207–211]. Second approach is the usage of ligand exchange mechanism by adding oil soluble ligand to synthesized particles mixture. By this method, not only are the dispersity and stability of magnetic particles improved, but also some new properties can be seen such as suitable biocompatibility, low increasing of particles size with shell layer, negligible decreasing of *M*_s_, the considerable usability in acidic, alkaline, aqueous, and oleic mediums, and the linking ability to biological target and biosensing targeting [207–211].

#### 4.1.2. Polymers

**Figure 15:**
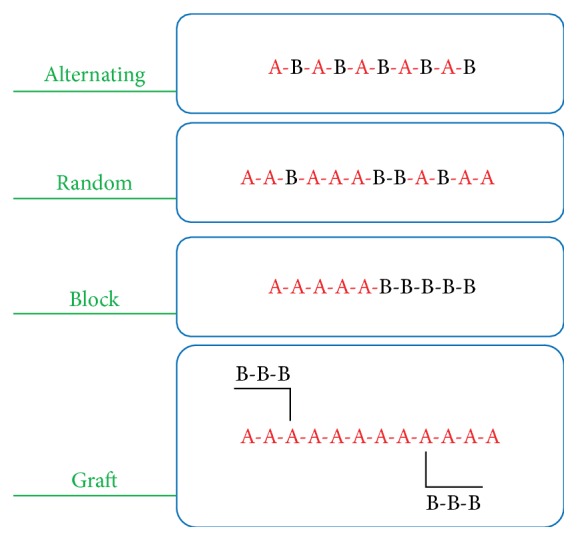
The schematic of some copolymer modes like alternating, random, block, and graft modes.

Generally, polymers have been formed by combination of small molecules and monomers to each other and creation of larger molecules [184]. The polymers are categorized into two different types, that is, homopolymers and copolymers, that both are utilized as functionalization and stabilization agents of magnetic particles. As a definition, homopolymers are the macromolecules with moderately high glassing temperature (*T*_g_) and brittle structure that are formed from polymerization of only single type monomers. These polymers are relatively smaller than copolymers, so they are known as simple polymers. Homopolymers are rarely used in stabilization of magnetite nanoparticles in bioapplications [212]. On the other hand, the macromolecules with low *T*_g_ and flexible structure that are polymerized based on two or more different monomers are called copolymers. These materials are the complex polymers and they can be divided into some modes including alternating, random, block, and graft copolymers.  Figure 15 shows the schematic of some copolymer modes.

Typically, polymers can lead to nanoparticles stabilization by their high repulsion forces. In fact, although polymers are caused to create electrostatic forces (repulsion) between particles, the major mechanism of stabilization is the induction of space repulsive forces due to volumes of polymers that are located on particles surface as coating layers [213–215].

In addition, owing to considerable biocompatibility properties of polymers, they are appropriate candidates to use in bioapplications (especially in vivo ones) [213–215]. Accordingly, polymers can be classified into natural (i.e., chitosan, dextran, and starch) and synthetic (i.e., PEG, PVA, and PMMA) polymers where in many bioapplications the natural ones are more suitable because of their better biocompatibility [64–70]. Each one of these polymers modifies the particles surface by their cationic or anionic nature. Some of the functional polymers and their properties are summarized in Table 5 [64–70].

Alongside the beneficial advantages of polymer coatings, these materials have some disadvantages; the first one is the average size increasing of nanoparticles around 40 to 60 nm after coating process that is undesirable status for numerous bioapplications. The second one is the falling of magnetic properties especially *M*_s_ of coated nanoparticles as a reason for the utilization of the large nonmagnetic molecules (polymers) as coating agents and thereby the augmentation of shell (polymer) to core (magnetite) diameter ratio [216–221]. According to these facts, the polymers are the appropriate coating for particles with high *M*_s_ or matrix structures (which contained many magnetic particles in polymer matrix). Polymer coating process can be done in two different methods including two-pot and in situ methods. The particles coating is carried out after synthesis process in two-pot method, while polymers can play critical roles as microcarriers for particles synthesis in one-pot method; thus, although the in situ methods usually have some complicities, they caused more suitable properties like particle dispersity, particles size, and even saturation magnetization of particles [216–221].

#### 4.1.3. Biological Molecules

According to importance of biocompatibility for many bioapplications especially in vivo ones, the biological molecules such as proteins, biotins, antibodies, and polypeptides have key roles as coatings [222–229]. In recent years, due to this considerable advantage, many works have been focused on the magnetic particles coating by utilization of biological molecules. For instance, albumin is used to coat the Fe_3_O_4_ nanoparticles in some works [149, 150, 184]. Usually, in order to utilize the biological coating, the surface of particles has been functionalized by surfactants and functional groups such as carboxyl and hydroxyl [222–229].

Main disadvantage of these molecules as coating agents is the increase of particles size (more than 100 nm) after coating process. For example, by using albumin molecules, the size of coated particles is increased to about 150 nm [149, 150].

### 4.2. Inorganic Coatings

**Figure 16:**
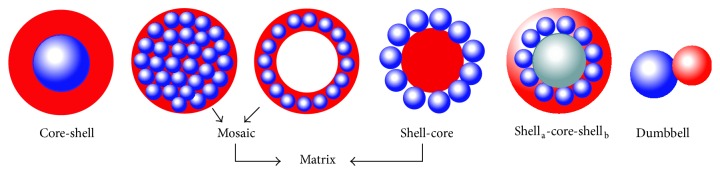
The schematic of some possible structures of inorganic coatings such as core-shell, matrix (mosaic), shell-core, and shell-core-shell structures [184].

In order to achieve suitable biocompatibility conditions, a range of inorganic materials can be used as a coating layer on magnetic particles. Typically, these materials have very considerable chemical stability and multifunctional properties [184]. For instance, the gold coating leads to creating the chemical stabilized biocompatible layer on the particles surfaces and it can play the essential role in optical hyperthermia applications. On the other hand, these materials have lower linking ability to other utilized materials like drugs, biotins, and so forth compared to organic coatings. Similar to organic materials, the inorganic coatings can form as various structures such as core-shell, matrix (mosaic), shell-core, and shell-core-shell structures.  Figure 16 shows the schematic of some possible structures [184].

Generally, inorganic coatings are classified into the two general categories, that is, metallic and ceramic coatings. Accordingly, the properties of these groups have been reviewed as the following.

#### 4.2.1. Metallic Coatings

Commonly, in order to improve the properties of magnetic nanoparticles, different metallic coatings including gold, silver, copper, platinum, palladium, and iron can be utilized [230–234]. As mentioned before, these materials caused the formation of biocompatible coating and creation of new applicable potentials. For example, the gold, silver, and copper nanocoatings show heat release by electromagnetic rays (infrared) promulgation as a basic mechanism in the optical hyperthermia applications [230–234]. In addition, these coatings can affect the magnetic properties (*M*_s_ and *H*_c_) of combined structures and their applications. Furthermore, usually the thickness of created coating layer by these agents is less than 10 nm and comparable with core diameter. Although gold, silver, and copper have an appropriate chemical stability, they demonstrate low *M*_s_ and *H*_c_ due to their diamagnetic behavior; thereby, they are suitable coatings to decrease *H*_c_ in the magnetic nanoparticles with high *M*_s_ [230–234]. Accordingly, unlike organic materials, the metallic coatings led to low rising of structure diameter; so, they are more proper cases to use in vivo applications such as capsulated particles for targeted drug delivery and hyperthermia [235–238]. Additionally, some magnetic metallic coatings like iron can be utilized to control the magnetic properties of coated structure owing to their special magnetic behaviors; that is, iron coating layers illustrate relatively higher *M*_s_ in comparison with the Fe_3_O_4_ core particles [235–238]. Moreover, these metallic coatings affect (commonly improving) the structure performance in some applications such as magnetic hyperthermia caused by development of new mechanisms for magnetic heat generation besides the relaxation loss mechanisms [235–238]. Generally, in order to coat the magnetic nanoparticles by metal agents, two methods are used including direct and indirect mechanisms. In direct method, the metallic ions are directly reduced on the bare surfaces of magnetic cores; hence, it caused noticeable decreasing of *M*_s_. Furthermore, by this coating method, the final formed structures have insignificant uniformity in size and morphology. The second one, indirect coating method, is carried out based on the utilization of activation groups like surfactants to link creation between functionalized cores surface and metallic ions as a coating layer. In contrast to direct methods, this mechanism can lead to forming the structures with more appropriate magnetic properties and shape uniformity, although their size is increased, commonly [230–238].

#### 4.2.2. Ceramic Coatings

The inorganic nonmetallic materials called ceramics are the very suitable candidates for use in many bioapplications. Generally, ceramics have excellent thermal, chemical, and functional stability due to their natures from the perspective of chemical compositions and crystallographic structures. These materials not only show considerable dispersibility and biocompatibility in many cases, but also can affect the electrical, optical, thermal, and especially magnetic behaviors of coated structures [184].

Owing to the superior dispersibility, biocompatibility, and chemical stability, carbon is a reasonable option for nanoparticles coating. However, carbon has very low *M*_s_ that is caused to decline the magnetic properties of coated structures [239, 240]. Silica (SiO_2_) is another ceramic that demonstrates excellent dispersibility, hydrophilicity, biocompatibility, and linking ability to other agents [136, 241–245]. Moreover, the process of nanoparticles coating by silica is relatively easy and low cost. On the other hand, silica coating has some disadvantages such as insignificant shape homogeneity of coated structures, low magnetic properties, high increasing of size, and wide size distribution of structures [136, 241–249].

Furthermore, different kinds of oxides such as CaO, ZnO, MgO, TiO_2_, CoO, and MnO can be used as a coating layer [250–261]. Additionally, some of these materials led to developing the functional properties of structures; that is, magnetic ferrites shells can uphold the magnetic properties of combined structures for bioapplications. For example, in recent years, Lee and colleagues suggested the new magnetic structures that are combined from magnetic ferrite (hard or soft) core and shell for the magnetic hyperthermia applications [262]. The different ferrites such as Fe_3_O_4_ (soft), CoFe_2_O_4_ (hard), MnFe_2_O_4_ (soft), and ZnFe_2_O_4_ (soft) can play the roles of core and/or shell parts, so that, typically, the soft phases increase *M*_s_ and the hard phases increase the anisotropy properties of structures that can lead to extreme improvement of the targeting process and heat generation in magnetic hyperthermia [262].

The overall comparison of critical properties of various coating materials is presented in Table 6.

## 5. Applications

In last decades, many applications have been established for magnetic nanoparticles especially magnetite ones. The unique magnetic properties as well as appropriate biocompatibility, dispersibility, and linking ability of these materials are caused to present different new bioapplications that can be categorized into two typical groups such as “in vitro” and “in vivo” applications [170]. In vitro applications included the activities that are performed in laboratory environment and outside of living organisms. The major ones of these applications are the magnetic detection and separation of cells, proteins, DNA, and so forth by use of superparamagnetic iron oxides nanoparticles (SPIONs) [170]. On the other hand, the considerable part of bioapplications is related to in vivo (refer to living samples) ones, that is, the disease diagnosis techniques like MRI, and the therapies methods such as magnetic hyperthermia and gene/drug delivery systems. In the following, some of the important ones are illustrated in detail.

### 5.1. Magnetic Resonance Imaging (MRI)

**Figure 17:**
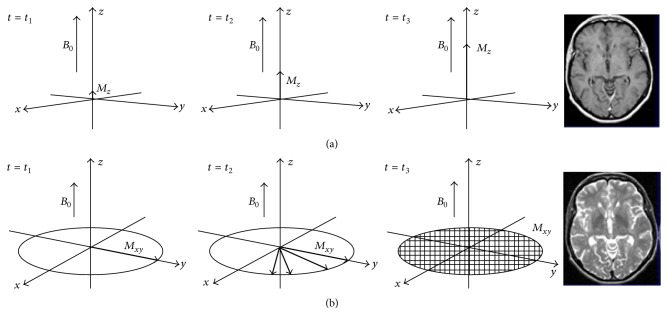
The schematic of *T*_1_ and *T*_2_ relaxations mechanisms. (a) The schematic of *T*_1_ recovery mechanism and (b) the schematic of *T*_2_ decay mechanism [190].

Nowadays, one of the most effective diagnostic techniques in medical fields is the magnetic resonance imaging that is briefly called MRI. In comparison with other medical imaging methods such as computed tomography (CT), positron emission tomography (PET), radiological imaging, and optical imagining, MRI is the strongest device for the investigation of various tissues and their difference to each other [136, 262–265]. The major advantage of MRI is the detectability of soft tissues like muscles, blood flow in vessels, and the density of each tissue in body [262–266]. In general, foundation of MRI mechanism is based on the alignment of unpaired magnetic spins of tissues to the applied magnetic field direction. In fact, many tissues of body contain more than 70% water (with different densities in various tissues); thus, according to the electron configuration of hydrogen atoms in the water molecules that is /1S^1^/, a large number of unpaired spins are variously reacted to the applied field in each tissue. In MRI device, the aligned spins are diverted from field direction by applying radio frequency (RF) signals and then they are returned to initial alignment by removing RF pulses [267]. The MR images are formed based on the returning time contrast of different tissues. These returns that are called relaxation are divided into the two modes: *T*_1_ and *T*_2_ relaxations [267]. *T*_1_ is related to the longitudinal relaxation of spins (spin-lattice coupling) and *T*_2_ is associated with the transverse relaxation (spin-spin coupling) [267].  Figure 17 shows the schematic of *T*_1_ and *T*_2_ relaxations mechanisms. In last decades, in order to improve the contrast of MR images, some materials with special magnetic properties were utilized as contrast agents that are caused to increase the number of reacted spins in tissues; thereby, the relaxation times and rates are reduced and augmented, respectively.

The mechanisms of these variations are formulated as follows:

(11) $$\begin{array}{c} R_{\text{CA}}=R_{0}+rC_{\text{CA}}, \end{array}$$

where *R*_CA_, *R*_0_, *r*, and *C*_CA_ are the relaxation rate after use of contrast agents, relaxation rate before use of contrast agents, relaxivity coefficient, and contrast agents concentration, respectively. This equation is usable for both *T*_1_ and *T*_2_ relaxation modes [267].

The contrast agents acted as an assistant of hydrogen spins for ordering by magnetic field applying. In fact, these materials can enhance the relaxation rates and MR image contrast between different tissues and organs especially between normal and abnormal ones. So, the quality of related diagnoses is improved [268–276].

Typically, according to effectiveness, the contrast agents are classified into two general types: positive and negative types. Positive contrast agents improved image contrast by increasing the *T*_1_ pulse intensity. The common positive contrast agents are the relatively lightweight compounds of gadolinium, manganese, and iron such as “gadopentetate dimeglumine,” “gadoteridol,” and “gadoterate meglumine.” Each one of these agents is suitable for specified tissues. On the other hand, the negative agents improved the image contrast by decreasing *T*_2_ pulse intensity. The superparamagnetic iron oxides nanoparticles (SPIONs) are the widespread samples of these agents such as some commercial ones like “Ferridex” and “Resovist” agents [277–284].

The utilization of SPIONs as MRI contrast agents has considerably speared in last decade, due to their most proper properties. For instance, compared to usual agents like gadolinium, SPIONs generally show higher *M*_s_ and lower *H*_c_ that led to offering noticeably superparamagnetic behavior [268–276]. Furthermore, not only are SPIONs nontoxic, but they also have more biocompatibility than other agents. In addition, SPIONs can be coupled with special materials like quantum dots and used in other supplementary diagnosis applications [268–276]. Moreover, owing to magnetic properties of SPIONs, these materials can be employed in other bioapplications (as a drug carrier in targeted drug delivery and heat generator in magnetic hyperthermia), simultaneously. In fact, SPIONs played roles as multifunctional agents. Accordingly, it can be concluded that SPIONs are the most appropriate candidates for MRI contrast agents in the future [262–284].

Generally, in order to improve the biocompatibility or/and functionalization of SPIONs, the formation of coating layer on particles surfaces is advantageous. The utilizable coating layers can be of different types such as polymers like dextran, metals like gold, and ceramics like SiO_2_ [184]. However, the common SPIONs that acted as a contrast agent today are coated by polymers and macromolecules, that is, dextran, citric acid, and carbohydrate chains [267].

### 5.2. Targeted Drug Delivery Systems

As mentioned before, in order to present new applications, the magnetic nanoparticles are coupled with different multifunctional materials and agents like gene or drugs. Accordingly, the benefit solution methods can be submitted to improve drug delivery systems and decrease side effects of therapy process [285–289]. In contrast to traditional chemotherapies, in these methods, the movement and distribution of drug in body are controlled and driven to special locations that contain tumors; so, they are called targeted drug delivery systems [285–289]. In recent years, although several targeted systems are suggested such as biosensing and modified enhanced permeability and retention based on size difference of tissues (EPR) methods, each of these systems has some shortcomings and limitations. For example, in common EPR techniques, not only is the drug delivery to the target a very time consuming process (tens of hours), but also the release rate of drug is relatively slow [290–292].

**Figure 18:**
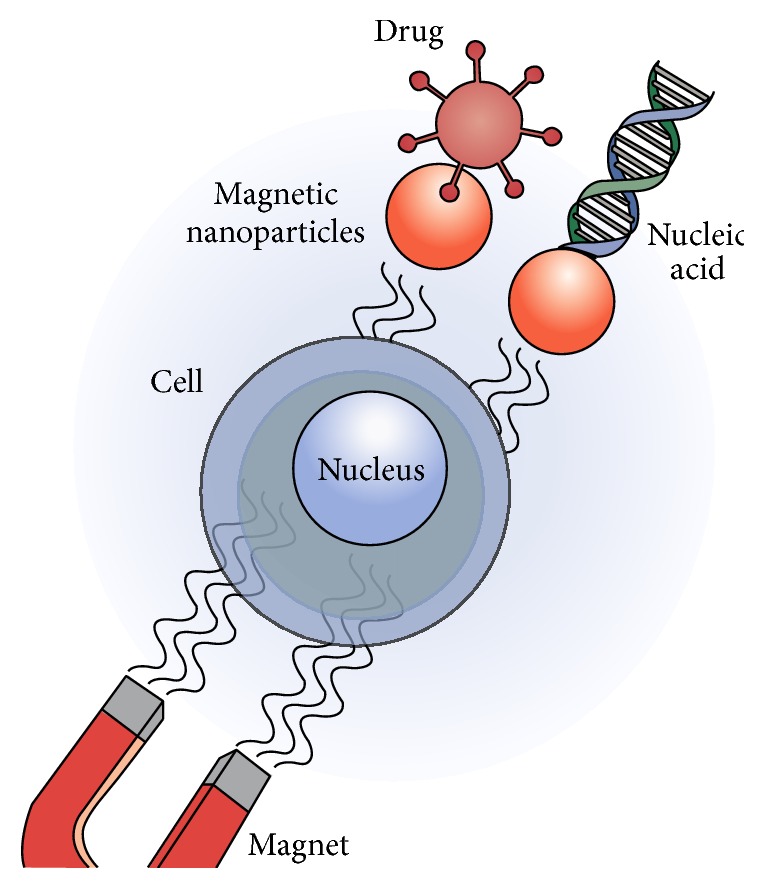
The schematic of magnetic targeted drug delivery mechanism [191].

As an alternative, the magnetic targeted drug delivery systems can improve these situations. In this method, the collections of drug/magnetic carriers (magnetic nanoparticles) are injected to blood circulation, and then by applying the external magnetic field that creates magnetic gradient in special spots of body (tumors), the drug/carrier sets are transferred and demonstrated in tumors [293–297].  Figure 18 shows the schematic of magnetic targeted drug delivery mechanism. Similar to other systems, the release techniques include enzymatic activity, osmolality changing, temperature altering, and especially pH varying [293–297].

In order to use magnetic nanoparticles in this system, they should have some terms. Size of particles is the most critical parameter since the small ones not only can move more freely in vessels, but also are detected by immune system rarely; thus, their retention time in blood is augmented [170]. Moreover, size of particles must be within the superparamagnetic behavior range to show proper performance. In fact, the minimum *H*_c_ and *M*_r_ amounts are the ideal magnetic conditions for these carriers. In addition, the higher amount of *M*_s_ helps in better targeting [170]. Furthermore, the biocompatibility of carriers is considerably important for this in vivo application.

The main advantages of magnetic targeted drug delivery systems include (1) decreasing of therapy side effects that arise from targeting process, (2) increasing of therapy efficiency due to reduction of drug losses, (3) ability to operate in conjunction with other methods for other related applications like MRI and hyperthermia, and (4) presenting new amendment aptitudes like magnetic thermal releasing mechanisms [293–297].

According to mentioned tips, Fe_3_O_4_ nanoparticles can be introduced as a very appropriate candidate for magnetic carriers of drugs owing to their biocompatibility and suitable magnetic properties [293–296].

In order to couple drugs and particles surfaces, different materials can be used such as ceramics, polymers, proteins, and surfactants. In fact, these functional groups like hydroxyl cause the biocompatibility, hydrophilicity, and linking ability properties of particles to be improved [184]. Additionally, some independent carriers like capsules and liposome can be utilized to movements of drug/magnetic particles complex. Generally, these capsulate systems have some benefits such as higher capacity of drug loading, better biocompatibility, and more suitable chemical and biological stability [184]. In recent years, many attentions have been focused on these combined systems. For instance, in 2010, Pradhan and colleagues used the combination of drug/Fe_3_O_4_ particles that is capsulated in nanoliposome for magnetic targeting drug delivery [298]. In another work, in 2012, Su and colleagues fabricated the coupling of drug/magnetite particles located in thermal sensitive polymer capsules to thermal release of drug [299]. However, in the majority of these researches, the magnetic properties of complex especially *M*_s_ are insufficient; hence, the movement and targeting process in body is relatively inappropriate [293–297].

### 5.3. Magnetic Hyperthermia

Thermal therapy called hyperthermia is one of the newest cancer therapies. In this method, the target tissues or tumor cells have been locally heated by different external sources [90, 170]. According to heat origins, hyperthermia is divided into some types such as electrical, optical, and magnetic hyperthermia. The intensity of generated heat and the targeting accuracy of heating are two critical terms of hyperthermia performance [90]. In comparison with other hyperthermia types, magnetic ones show more appropriate conditions due to their sufficient produced heat and proper targeting precision.

**Figure 19:**
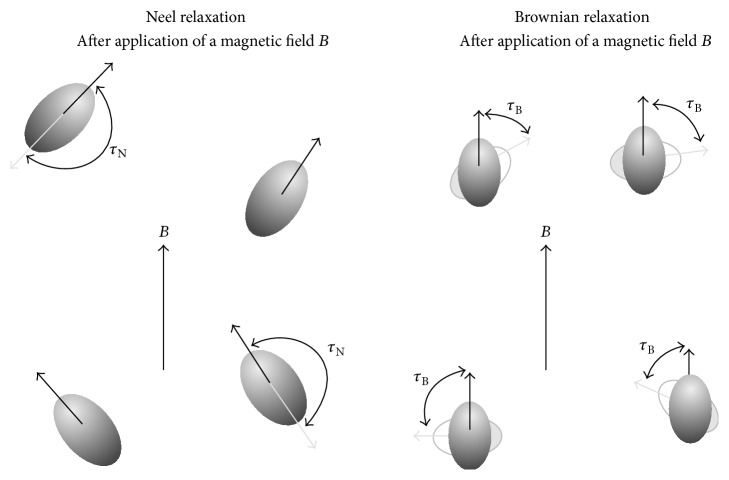
The schematic of magnetic relaxation mechanisms such as Neel and Brownian relaxation mechanisms [90].

In hyperthermia based on use of magnetic nanoparticles, like targeting drug delivery systems, the particles are injected into the body and then moved in blood circulation and transferred to tumors location by following applied DC magnetic field. In the next step, these particles can act as heat generators in the tissues by applying alternating magnetic field. The dominant mechanism of thermal losses (heat generate) of magnetic nanoparticles is the relaxation process that has two states. In the first state, the thermal loss occurred by rotation of magnetic moments in particles under AC magnetic field called Neel relaxation. The second one is the rotation of particles in the surrounding environment that is known as Brownian relaxation and associated with fluid viscosity [90].  Figure 19 indicates the schematic of these relaxation mechanisms.

The Neel and Brownian relaxation times are calculated based on the following equations [300–304]:

(12) τB=3VhydKBT×η,τN=τ0exp⁡KVMKBT,τeff=τB×τNτB+τN,

where *τ*_B_, *τ*_N_, *τ*_eff_, *τ*_0_, *K*, *V*_*M*_, *K*_B_, and *V*_hyd_ are the Brownian relaxation time, Neel relaxation time, effective relaxation time, constant time equal to 10^−9^ seconds, magnetic anisotropy constant, particle volume, Boltzmann constant, and hydrodynamic volume of particle, respectively.

According to these equations, it is clear that the smaller particles generate lower losses while the magnetic anisotropy in Neel relaxation and viscosity in Brownian one have positive effects in thermal losses. Considering that the viscosity of fluid is relatively fixed, the Neel relaxation is the leading mechanism; thus, in magnetic hyperthermia application, the utilized particles should have high anisotropy [300–303].

Typically, in order to compare the operation of different materials in hyperthermia application, the criterion for thermal loss intensity in determined time can be defined. This standard is called “specific loss power” (SLP) and it is equal to produced heat per unit volume of particles; so, the higher amount of SLP is more suitable. SLP amount depends on various parameters, that is, particle size and distribution, magnetic anisotropy, *M*_s_, and surface conditions [300–303]. For the other interpretation, SLP is expressed as another term which is called “specific absorption rate” (SAR) with the following formula:

(13) $$\begin{array}{c} \text{SAR}=C\times \left(\;\frac{\Delta T}{\Delta t}\;\right)\times \left(\;\frac{1}{m_{\text{ferrite}}}\;\right), \end{array}$$

where *C*, (Δ*T*/Δ*t*), and *m*_ferrite_ are the specific heat coefficient of nanofluid, the initial slope of temperature-time curve, and the mass of ferrite, respectively [305].

Like other in vivo applications, the biocompatibility and biological stability of used particles in hyperthermia are extremely important. Accordingly, although some ferrite structures like CoFe_2_O_4_ have considerably high anisotropy, they are weakly biocompatible materials; thereby, the iron oxides and especially magnetite nanoparticles are introduced as most excellent candidates because of their biocompatibility, chemical stability, and proper magnetic properties [300–303]. Among various types of iron oxides, Fe_3_O_4_ is more notable due to its higher *M*_s_ and anisotropy amounts. Furthermore, the utilization of different coating materials such as ceramics, polymers, surfactants, and biomolecules can modify particles surface and improve their required properties [300–305].

**Figure 20:**
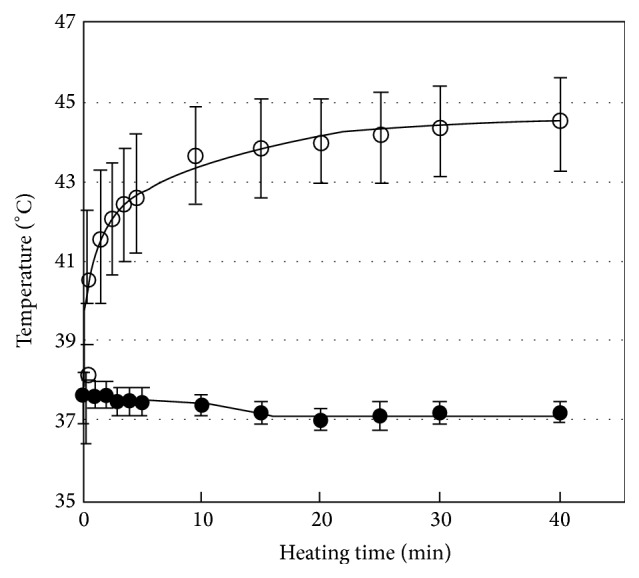
The difference of temperature increasing between target tissue, which is containing Fe_3_O_4_ particles (marked with solid circles), and another one, without these particles (marked with hollow circles). Based on this figure, the presence of magnetic particles causes targeted hyperthermia [192].

Although the magnetite particles had been used in magnetic hyperthermia systems for the first time in the 1990s, many related researches have been done in recent years [306]. For example, in 2003, Hamaguchi and colleagues produced the hyperthermia systems by combination of Fe_3_O_4_ particles with size of 10 nm that were located in liposome with size of 100 nm [192].  Figure 20 shows the difference of temperature increasing between target tissue (i.e., containing Fe_3_O_4_ particles) and another one (without these particles).

**Figure 21:**
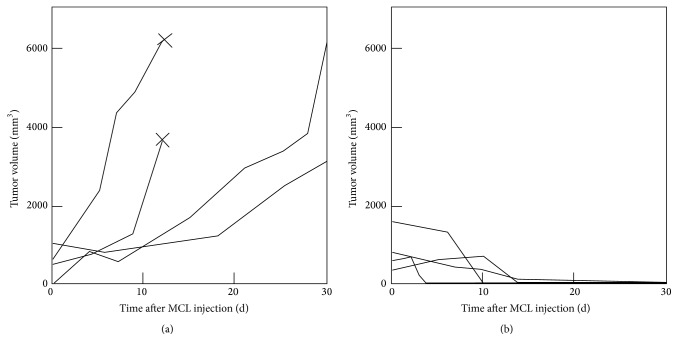
The effect of applying magnetic hyperthermia with magnetite particles in hamster samples after 30 days (120 kHz AC magnetic field, 90 min per day). In case (a), without hyperthermia treatment, the tumors volume is increased while in case (b) the volume of treated tumors by hyperthermia is obviously decreased [193].

Based on this figure, the presence of magnetic particles causes targeted hyperthermia; so, the side effects of this therapy are significantly reduced. In other works, as seen in Figure 21 by applying magnetic hyperthermia with magnetite particles in hamster samples, the volume of treated tumors is obviously decreased [193]. Moreover, Gonzales and Krishnan found that the optimum size of Fe_3_O_4_ particles for hyperthermia is about 11 nm since the smaller particles generate insufficient amount of heat [307]. In 2011, Lee and colleagues suggested new ferrite-ferrite structures like MnFe_2_O_4_-CoFe_2_O_4_ that caused impressive augmentation of SLP amounts [262].

On the other hand, in some researches these hyperthermia systems were coupled with other related applications like MRI and targeted drug delivery. For instance, in recent years the magnetic hyperthermia and targeted drug delivery systems are successfully combined with each other [308]. Furthermore, in 2013, Clares and teams introduced combination of hyperthermia system and MRI contrast agents by utilization of liposomal carriers containing Fe_3_O_4_ nanoparticles with size of 20 nm [309].

Typically, the main effective parameters of magnetic particles in some bioapplications are seen in Table 7.

## 6. Conclusion

In this work, the attentions were focused on roles of magnetic nanoparticles especially Fe_3_O_4_ in some bioapplications such as MRI contrast agents, targeted drug delivery, and magnetic hyperthermia systems. Based on FDA licenses, magnetite nanoparticles show suitable biocompatibility, dispersity, and the chemical and biological stability. Moreover, these particles have excellent magnetic properties like relatively high *M*_s_ caused by unique variations in structures when their size is decreased to nanometric scales. Considering the significance of magnetic properties, the various methods and devices, that is, VSM, SQUID, and FAGM, are employed for their measurements. However, the recognition of measurements errors can be very useful to improve process accuracy.

In order to achieve appropriate performance, the presentation of superparamagnetic behaviors is necessary which occurred in size range of about 20 to 30 nm for the majority of ferrites; thus, the size of particles has significant importance. The different synthesis techniques can affect various properties particularly particle size and distribution. Although thermal decomposition and hydrothermal methods have more complicity related to other methods like coprecipitation, they indicate lower size and distribution with superior magnetic properties. In addition, the used coatings not only improve the biocompatibility and dispersity, but also develop the linking ability of particles surface for some applications. Depending on the usage, different coatings such as surfactants, polymers, biomolecules, metals, and ceramics cause a range of effects on properties. Typically, surfactants and some biomolecules can cause smaller particles with more proper magnetic characteristics.

Generally, in recent years, many bioapplications have been suggested based on these nanoparticles such as in vitro and in vivo ones. According to application type, the required situations and related properties of particles must be designed. For instance, in magnetic hyperthermia, the utilized nanoparticles should have high magnetic anisotropy, while, for targeted drug delivery systems, it is unessential point. Considering the recent studies on the combination of various applications like MRI contrast agents, targeted drug delivery, and hyperthermia, it can be hoped that, in the nearest future, magnetic nanoparticles pose great developments in the medicine and bioscience fields.

## Figures and Tables

**Table 1:** The list of some critical magnetic parameters with their units in SI system.

Parameter	Symbol	Unit (SI)
Magnetic moment	*m*	Am^2^
Magnetization	*M*	A/m
Magnetic field	*H*	A/m
Magnetic flux density	*B*	T (tesla)
Magnetic polarization	*J*	T (tesla)
Curie temperature	*T*_C_	K
Blocking temperature	*T*_B_	K

**Table 2:** The rough comparison of important parameters of different measurement methods like measurable properties and sample conditions.

Method	Measurable properties	Sample conditions	
		Phase	Geometry
VSM	*H*, *B*, *H*_c_, *B*_r_, *J*, *μ*, *T*_C_	Amorphous, polycrystalline, nanocrystal	Bulk, powder, thin film
SQUID	*H*, *B*, *H*_c_, *B*_r_, *J*, *μ*, *T*_C_	Amorphous, polycrystalline, nanocrystal	Bulk, powder, thin film
Torque magnetometer	*K* (anisotropy constant)	Amorphous, polycrystalline, nanocrystal	Bulk, nanoparticle, single crystal
Helmholtz coil	*J*	Polycrystal	Bulk
Coercimeter	*H*_c_	Ferromagnetic metal	Bulk

**Table 3:** The critical size of transform to superparamagnetic state for some common magnetic materials.

Magnetic material	Fe_3_O_4_	CoFe_2_O_3_	NiFe_2_O_3_	MnFe_2_O_3_
*D*_SP_ (nm)	23	15	27	25

*D*_SP_: critical size of transfer to superparamagnetic state.

**Table 4:** The comparison of key properties of different synthetic methods [61–63].

Synthetic method	Synthesis	Reaction temp. (°C)	Reaction time	Solvent	Surface-capping agents	Size distribution	Shape control	Yield
Coprecipitation	Very simple	20–90	Minutes	Water	During/after reaction	Relatively narrow	Not good	High
Microemulsion	Complicated	20–50	Hours	Organic agents	During reaction	Relatively narrow	Good	Low
Thermal decomposition	Complicated	100–320	Hours-days	Organic agents	During reaction	Very narrow	Very good	High
Hydrothermal	Simple	200–250	Hours-days	Water-ethanol	During reaction	Very narrow	Very good	Medium

**Table 5:** Some of the functional polymers and their important properties [64–70].

Polymer nature	Type	Important properties
Natural	Dextran	Good stability, biocompatible
	Chitosan	Hydrophilic, biocompatible, suitable for gene delivery
	Starch	Biocompatible, suitable for MRI contrast and drug delivery
	Gelatin	Biocompatible, hydrophilic

Synthetic	PEG	Good stability, hydrophilic, biocompatible
	PVA	Improved monodispersibility
	PLA	Biocompatible, biodegradability
	PMMA	Suitable for drug delivery and cell separation

**Table 6:** The overall comparison of critical properties of different coating materials.

Coating materials	Properties					
	Biocompatibility	Chemical stability	Dispersibility	Size	Magnetic properties	Linking ability
Organic						
Surfactants	High	Medium	High	Low	Medium/high	Very high
Polymer	High	Medium	High	Very high	Low/medium	Very high
Biomolecules	Very high	High	High	Very high	Low	Very high
Inorganic						
Metallic	Medium/high	Medium/high	High	Low	Medium/high	High
Ceramic	High	Very high	Very high	Medium/high	Medium/high/very high	Medium

**Table 7:** The main effective parameters of magnetic particles in some bioapplications.

Application	Parameters					
	Size	Biocompatibility	*M*_s_	Superparamagnetism	Mag. anisotropy	Surface linking ability
MRI	Yes	Yes	Yes	Yes	No	No
Targeted drug delivery	Yes	Yes	Yes	Yes	No	Yes
Mag. hyperthermia	Yes	Yes	Yes	Yes	Yes	No

## Additional Points

The paper highlights are (i) inclusive surveys on importance of magnetic nanoparticles for bioapplications, (ii) evaluation of synthesis techniques of magnetic nanoparticles for bioapplications, (iii) study of surface modification treats of magnetic nanoparticles for bioapplications, (iv) investigation of critical properties of magnetic nanoparticles for bioapplications, (v) the review containing essential magnetic properties and their measurement methods.
